# Coconut Shell-Derived Activated Carbons: Preparation, Physicochemical Properties, and Dye Removal from Water

**DOI:** 10.3390/molecules31020263

**Published:** 2026-01-12

**Authors:** Vanda María Cachola Maldito Lowden, María Francisca Alexandre-Franco, Juan Manuel Garrido-Zoido, Eduardo Manuel Cuerda-Correa, Vicente Gómez-Serrano

**Affiliations:** Departamento de Química Orgánica e Inorgánica, Facultad de Ciencias, Universidad de Extremadura, Avenida de Elvas s/n, 06006 Badajoz, Spain; vanda_cachola@hotmail.com (V.M.C.M.L.); emcc@unex.es (E.M.C.-C.); vgomez@unex.es (V.G.-S.)

**Keywords:** activated carbons, coconut shell waste, valorization, physical activation, chemical activation, dye adsorption, green chemistry

## Abstract

Valorizing coconut shell waste as a renewable lignocellulosic precursor offers a sustainable route to produce high-performance activated carbons for wastewater treatment. In this study, coconut shells were transformed into activated carbons through physical activation (air, CO_2_, steam) and chemical activation (H_3_PO_4_, ZnCl_2_, KOH), allowing direct comparison of how each method influences porosity and surface chemistry. Among the physically activated samples, steam activation produced the best material, A-ST, with S_BET_ = 738 m^2^ g^−1^, V_mi_ = 0.38 cm^3^ g^−1^ and V_me_ = 0.07 cm^3^ g^−1^. KOH activation yielded the top-performing carbon, A-KOH, achieving S_BET_ = 1600 m^2^ g^−1^, V_mi_ = 0.74 cm^3^ g^−1^, and V_me_ = 0.22 cm^3^ g^−1^. Adsorption tests with methylene blue, methyl orange, and orange G showed a clear link between physicochemical features and dye uptake. A-ST and A-KOH exhibited the highest capacities due to their wide micro–mesoporosity and favorable surface charge at the adsorption pH. In both cases, methylene blue was most strongly retained, confirming that large aromatic cations benefit from π–π interactions with graphene-like layers and easy micropore access. Overall, the results demonstrate that coconut-shell valorization is maximized when activation enhances both porosity and surface chemistry, enabling the production of tailored sorbents for the efficient removal of organic contaminants.

## 1. Introduction

Activated carbon is one of the most widely used adsorbents for water treatment and environmental remediation due to its high porosity, large specific surface area, and the versatility associated with surface functional groups. However, its conventional production relies predominantly on non-renewable precursors—such as mineral coals, peat, or petroleum-derived residues—and involves energy-intensive processes and extensive use of chemical reagents, which lead to significant environmental impacts throughout its life cycle. In this context, there is a growing interest in developing more sustainable synthesis routes based on renewable feedstocks and agro-industrial wastes.

Coconut shell (CS) is an agro-industrial residue available in tropical and subtropical regions. Global coconut production exceeded 62 million tons in 2022, mainly in Indonesia, the Philippines, and India [1], generating large volumes of lignocellulosic by-products such as the husk and the shell. In many cases, these residues are managed through open-air burning or uncontrolled disposal, contributing to greenhouse gas emissions and local impacts on air and soil quality. The transformation of CS into activated carbon represents an efficient valorization strategy, converting a low-value waste into a technologically relevant adsorbent while reducing dependence on fossil-based precursors and improving the sustainability of agro-industrial waste management.

Activated carbons derived from CS typically exhibit high mechanical hardness, low ash content, and strong microporosity, properties that reflect the lignocellulosic nature and high fixed-carbon fraction of the precursor [2,3]. These characteristics make CS-based activated carbons particularly suitable for the removal of small organic pollutants and certain metal ions from aqueous media. However, the efficient adsorption of large organic molecules—such as many industrial synthetic dyes—requires the development of sufficient mesoporosity to facilitate access and diffusion within the porous network [4,5,6]. Therefore, the optimization of the micro-/mesopore balance, combined with precise control of the surface chemistry (acidity/basicity, oxygen-containing groups, heteroatom incorporation), is a key factor to maximize their performance in water treatment applications.

Activated carbon production from CS has been approached through physical, chemical, and combined activation processes, employing activating agents such as CO_2_, steam, KOH, NaOH, ZnCl_2_, H_3_PO_4_, or H_2_SO_4_. Recent studies report highly heterogeneous textural properties, with specific surface areas ranging from less than 20 m^2^ g^−1^ to over 2800 m^2^ g^−1^, highlighting the strong influence of the activation protocol, the activating agent, and experimental conditions (precursor/activating agent ratio, temperature, residence time, atmosphere, washing stage). In general, physical activation (CO_2_ or steam) produces predominantly microporous structures, whereas chemical activation enables greater control over both mesopore development and surface functionalities. Combined activation strategies (chemical and physical) have emerged as a promising approach for producing hierarchical micro- and mesoporous structures with high adsorption capacity and fast adsorption kinetics [7,8,9]. A comparative summary of the most relevant studies on CS-derived activated carbons—including preparation routes, activating agents, and key textural parameters—is presented in Table 1, serving as a contextual framework for the present research.

In parallel, regulatory and societal pressure on the management of industrial wastewater has increased markedly over the past decade. The textile sector, heavily dependent on dyeing and finishing processes, is recognized as a major contributor to industrial water pollution due to the release of dye baths containing unfixed dyes, salts, surfactants, and auxiliary chemicals [10]. These effluents exhibit high organic load, persistent coloration, and, in many cases, acute and chronic aquatic toxicity, which make them resistant to conventional treatments such as coagulation-flocculation, biological degradation, or membrane filtration [11]. In addition to reducing light penetration and altering photosynthetic processes in aquatic ecosystems, numerous dyes and their degradation products show carcinogenic, mutagenic, or endocrine-disrupting effects [12,13]. Therefore, their efficient removal from water is considered an environmental priority within the Sustainable Development Goals framework.

In this context, low-cost adsorbents derived from lignocellulosic wastes—particularly CS-based activated carbons—have received increasing attention as alternatives or complements to advanced treatment technologies such as oxidation, photocatalysis, or membrane systems, due to their high efficiency, operational simplicity, and regeneration potential. Several studies have demonstrated that CS-derived activated carbons can achieve high adsorption capacities for basic and reactive dyes, often following Langmuir and Freundlich isotherms and exhibiting adsorption kinetics well described by pseudo-second-order models [14]. However, the best performances are generally obtained for dyes of small or moderate molecular size, whereas adsorption capacity and rate decrease markedly for bulky dye structures when the adsorbent exhibits predominantly microporous characteristics. This behavior highlights the need to design adsorbents with hierarchical porosity and tailored surface chemistry adapted to the structural and charge properties of the target dyes.

**Table 1:** A literature review of the methods used in the preparation of activated carbon from CS.

Activation Method	Activating Agent	Impregnation/Ratio (%), T (°C), t (h)	Carbonization * Atmosphere, T (°C), t (h)	Activation/T (°C), t (h)	S_BET_/m^2^ g^−1^	Ref.
Physical	CO_2_			800, 1	720	[15]
Chemical and Physical	KCl, KNO_3_, KOH, K_2_CO_3_, K_3_PO_4_/CO_2_	5.5, 70			400–1550	[15]
Physical	CO_2_			800		[16]
Chemical and Physical	KOH, CO_2_ H_3_PO_4_, CO_2_	5.5 3.0			751–1360	[16]
Chemical	KOH	0.25–0.75, 800, 2	400–800, 1–3		1186–245	[17]
Physical	CO_2_		900, 1	900, 1–1.5	>350~1800	[18]
Pyrolysis	N_2_		250–850, 1		663	[19]
Physical	O_2_ (air)			800	700	[20]
Physical	H_2_O (steam)			900, 0.5	2114	[21]
Chemical	ZnCl_2_	1:1, 80, 14	500, 3		266	[21]
Physical	H_2_O (steam)			1000		[22]
Chemical	ZnCl_2_	0.1–0.5, 24	700–800, 0.5			[23]
Physical	CO_2_			600, 2	186	[24]
Chemical and Physical	KOH, CO_2_	1:1, 110, 12	700, 1	850, 2	1026	[25]
Physical	CO_2_			900, 1	1667	[26]
Physical	CO_2_			900, 1.5	2000	[27]
Chemical	NaOH	1:1–3:1	130–500, 1–2		783–2825	[28]
Chemical	H_2_SO_4_	12 M, 300, 1	250–850, 1		16	[29]
Physical	CO_2_		850, 2	750, ≠t		[30]
Chemical	H_3_PO_4_, ZnCl_2_	2 mg/g, 85, 2	2			[30]
Chemical and Physical	ZnCl_2_ y CO_2_					[31]
Physical	CO_2_			850, 5		[32]
Pyrolysis	N_2_		850, 0.5		229	[33]
Physical	CO_2_			800, 1	1327	[34]
Chemical	H_3_PO_4_	1:1	500, 2		483	[35]
Chemical	KOH	1:3			1650	[36]
Chemical	H_2_SO_4_	1:2 (6N), 24	220, 2		509	[37]
Chemical and Physical	KOH, CO_2_	1:5		700, 2	478	[38]
Chemical	H_2_SO_4_	1: 3 (2N), 24			818	[39]
Chemical	NaOH	1:1, 3	400, 1	1000, 5	520	[40]
Physical	H_2_O (steam)			800, 0.5	1011	[41]
Chemical	ZnCl_2_	1:1	500, 1		1223	[41]
Chemical	H_2_SO_4_				422	[42]
Chemical	NaOH	1:1, 105, 4	600, 1		876	[43]
Chemical and Physical	ZnCl_2_ y CO_2_	4:1		800, 2	1796	[44]
Chemical	KOH		400, 4		250	[45]
Chemical	H_2_SO_4_	H_2_SO_4_, 24	500, 2			[46]
Pyrolysis			900, 6		1194	[47]
Chemical	KOH	1:4	700, 3		1148	[48]
Chemical	K_2_SO_3_				1430	[49]
Physical	CO_2_			800, 1	362	[50]
Chemical and Physical	KOH, CO_2_			750		[50]
Chemical	CH_3_CO_3_H	100, 3			411	[51]
Chemical and Physical	NaOH y CO_2_	3:1		700, 1	2056	[52]
Physical	Steam			850, 1	1042	[53]
Chemical	HNO_3_, NaOH, KMnO_4_, FeSO_4_				≤982	[54]
Pyrolysis			Hydrothermal 180, 12		287	[55]
Chemical	KOH	(1:1, 1:2, 1:3), 90, 2		800, 4	1178–1567	[55]
Physical	CO_2_			Microwave	675	[56]
Chemical	Fe_2_O_3_ Fe_2_O_3_ + HCl		950–1050, 1		250–1200	[57]
Chemical	NaOH, ZnCl_2_, H_3_PO_4_	1:4, 85, 2	600, 3		516, 42, 23	[2]
Physical	CO_2_			Microwave	626	[58]
Chemical	KOH	1:1, 700–800			973–1726	[59]
Chemical	KOH KOH + GO		Hydrothermal 500, 2		478, 278	[60]
Chemical	NaOH, H_2_SO_4_	2N, 12	600, 2		345, 40	[61]
Physical	CO_2_		800, 4	800, 4	602	[62]
Chemical	ZnCl_2_	2:5, 80, 4		600	1391	[62]
Chemical	ZnCl_2_, H_3_PO_4_	(1:1, 1:3)		600–900, 1		[63]

* Thermal carbonization treatments have generally been carried out under a N_2_ atmosphere.

The adsorption of synthetic dyes onto activated carbons is driven by a combination of physical and chemical interactions, including electrostatic attraction, π–π interactions between aromatic rings and graphitic domains, hydrogen bonding, and, in certain cases, n–π interactions or coordination with heteroatoms [64,65]. The relative contribution of each mechanism depends on factors such as solution pH, point of zero charge (pH_pzc_) of the adsorbent, distribution of surface functional groups, presence of heteroatoms in the carbon matrix, and the electronic structure of the dye. Controlling these parameters through the selection of the activation protocol—and, if required, post-functionalization treatments—provides a rational route to improve adsorption capacity and selectivity toward specific dye families.

In addition to organic dyes, coconut-derived materials have been reported as effective adsorbents for emerging water pollutants such as nanoparticles. Coconut husk, which is rich in lignin and cellulose and exhibits a porous and structurally stable framework, has been shown to efficiently adsorb nanomaterials, including nanocrystalline metal oxides, from aqueous solutions. In this context, the activated carbons prepared in the present work from coconut shell display highly developed microporosity and large specific surface areas, characteristics that are particularly favorable for the adsorption of small-sized species. Although nanoparticle removal was not directly investigated here, the textural properties demonstrated for these carbons suggest their potential applicability in the adsorption of nanoparticles, in agreement with recent reports on coconut-based adsorbents [66].

In this study, two activation strategies, namely, physical and chemical, are systematically investigated for the preparation of activated carbons derived from coconut shell, with the aim of determining how the preparation route influences the porous structure and surface chemistry of the material. Particular attention is devoted to the micro-/mesopore balance and to the modification of surface functional groups associated with each activation protocol. Furthermore, the adsorption performance of the resulting materials is evaluated toward three organic dyes selected as model contaminants, representative of different chemical families with contrasting molecular sizes and charges (e.g., cationic thiazine dyes and anionic azo dyes) widely used in industry. This comparative approach enables clarification of the relationship between preparation route, physicochemical properties, and adsorption efficiency toward large organic molecules in aqueous solution, providing criteria for the design of sustainable carbon-based adsorbents specifically targeted to the treatment of industrial dye-containing wastewater.

Coconut shell is widely recognized as an excellent precursor for activated carbon production due to its high fixed carbon content and its tendency to develop predominantly microporous structures. While this feature is advantageous for the adsorption of small molecules, it may represent a limitation for the removal of solutes with larger ionic or molecular dimensions, such as many industrial organic dyes, whose access to narrow micropores is sterically hindered. Moreover, insufficient mesoporosity can lead to slow adsorption kinetics, reducing the practical efficiency of coconut-shell-derived carbons in wastewater treatment applications. In this context, the novelty of the present work lies in the systematic comparison of traditional physical and chemical activation routes—using the most commonly applied activating agents—to elucidate how each preparation strategy modifies the micro- and mesoporous texture and surface chemistry of coconut-shell-derived activated carbons.

Physical activation is the most common method of preparing activated carbon. Basically, it consists of two successive stages, the first one of carbonization (i.e., heat treatment of a given starting material in an inert atmosphere, usually of N_2_) and the second one of activation (i.e., subsequent heated treatment of the obtained carbonized product in an oxidizing/gasifying atmosphere of air, CO_2_, or steam). Nevertheless, activated carbon can also be prepared in a single stage by direct activation of the starting material in air. Regardless of the method, porosity is developed through partial gasification of the carbon matrix without prior chemical modification of the precursor. In contrast, chemical activation involves carrying out an initial impregnation stage in which the raw precursor is brought into contact with the chemical activating agent (e.g., ZnCl_2_, H_3_PO_4_, or KOH), usually in aqueous solution, before the carbonization/activation stage/s. This impregnation promotes dehydration, bond cleavage, and chemical templating effects during subsequent heat treatment, leading to more pronounced pore development and modification of surface chemistry.

By directly correlating these physicochemical features with the adsorption behavior of three structurally distinct organic dyes, differing in size, charge, and polarity, this study provides new insight into the structural limitations of coconut-shell-based carbons and demonstrates how appropriate activation strategies can overcome these constraints to enhance adsorption capacity and kinetics.

## 2. Results and Discussion

### 2.1. Physicochemical Characterization of Coconut Shell Precursor

Elemental analysis (Table 2) showed that CS is rich in carbon and hydrogen, and contains very low amounts of sulfur and nitrogen, which confirms its suitability as a precursor for activated carbon production. In addition, the ash content was extremely low (0.45%), a desirable feature to minimize the negative impact of inorganic matter on catalytic and adsorption processes. It should be noted, however, that the subsequent pyrolysis/activation steps are expected to decrease the overall sample mass and concomitantly increase the relative concentration of inorganic species in the carbonized and activated products.

**Figure 1:**
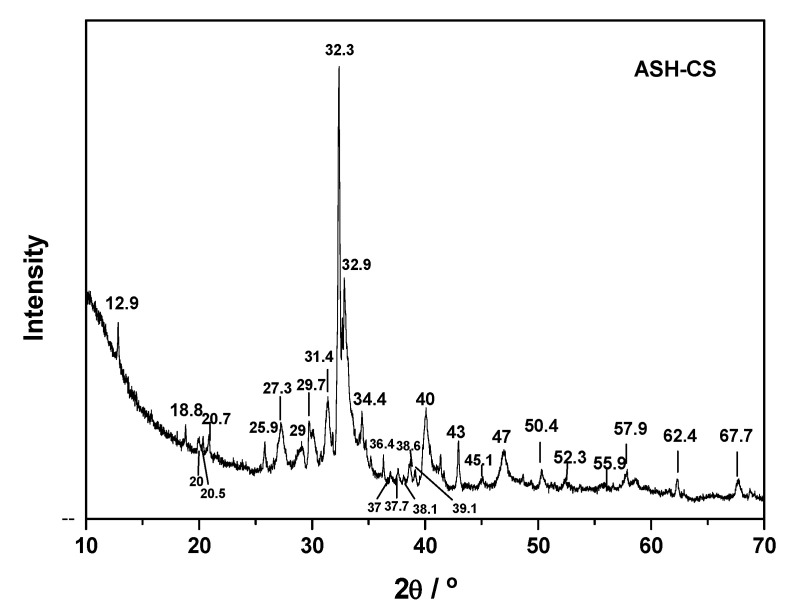
X-ray diffractogram of coconut shell ashes.

The ashes obtained after CS incineration were analyzed by wavelength-dispersive X-ray fluorescence (WDXRF) and X-ray diffraction (XRD). WDXRF (Table S1, Supplementary Materials) revealed that the major oxides were K_2_O, Na_2_O, SiO_2_, and CaO, in line with the typical composition of biomass ashes and with the relative abundance of these elements in the Earth’s crust, from which plants take up nutrients. Some discrepancies with previous reports—where significant amounts of Fe_2_O_3_ and Al_2_O_3_ were found—can reasonably be attributed to differences in the mineralogical composition of the soils where the coconut palms were cultivated [67]. The XRD pattern of CS ash (Figure 1) exhibited multiple diffraction peaks of varying width and intensity, evidencing both chemical complexity and a certain degree of crystallinity. Low-intensity peaks in the 2θ ranges 25–32° and 41–45° were tentatively assigned to K_2_O and Na_2_O, while a peak at around 2θ ≈ 21° was ascribed to SiO_2_. The two most intense peaks at 2θ ≈ 32–34° were compatible with the presence of Ca_2_(Al_x_Fe_1−x_)_2_O_5_ (ferrite) and Ca_3_(Al_2_O_6_) (orthorhombic aluminate) [68].

**Figure 2:**
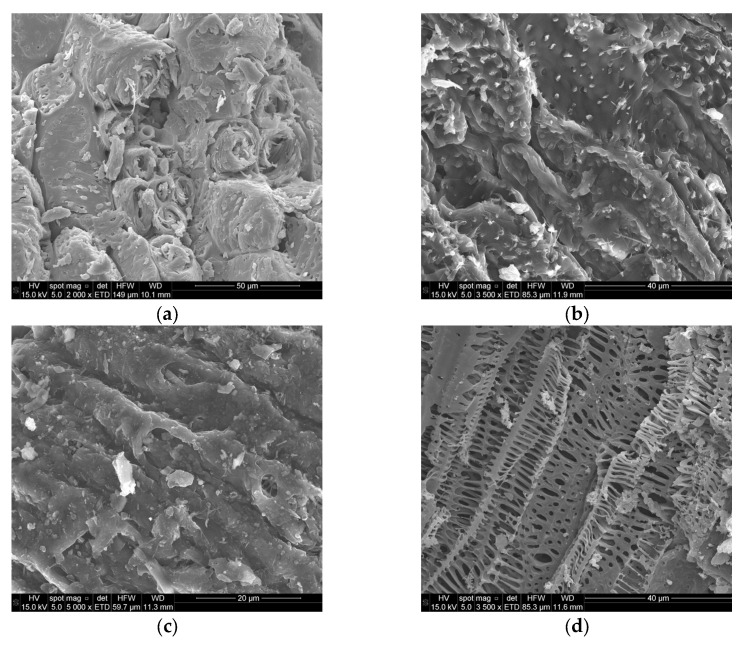

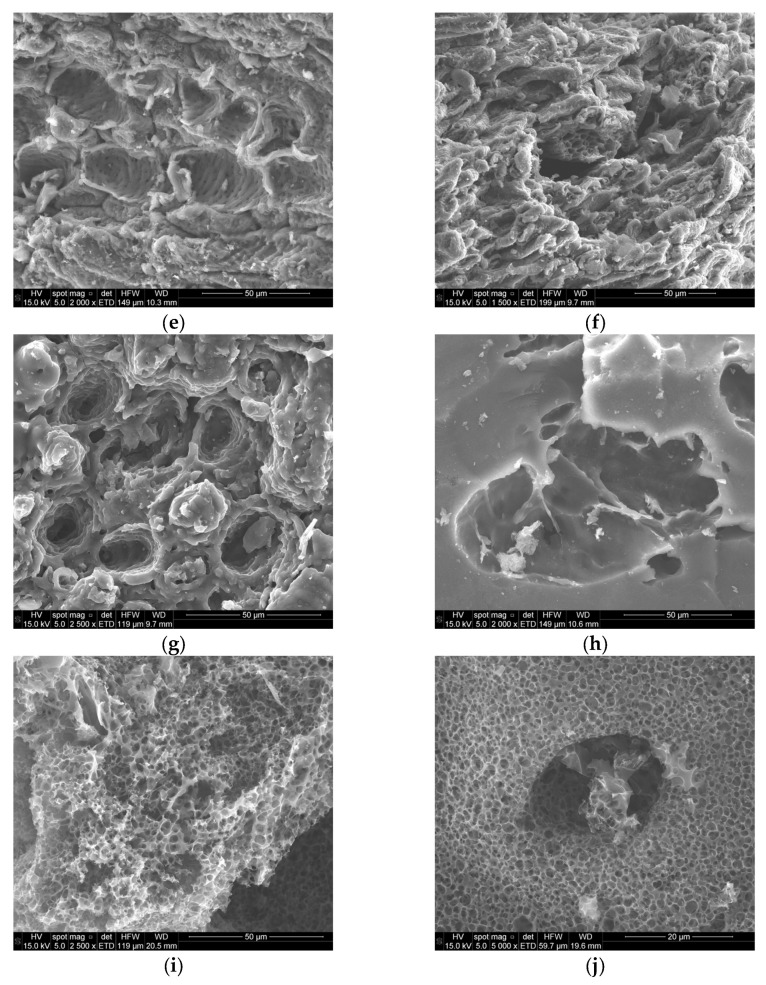
SEM micrograph. Samples: (**a**) CS; (**b**) C-600; (**c**) C-900; (**d**) A-O_2_; (**e**) A-CO_2_; (**f**) A-ST; (**g**) A-ZnCl_2_; (**h**) A-H_3_PO_4_; (**i**) A-KOH; (**j**) A-KOH zoom.

The morphology of CS was examined by scanning electron microscopy (SEM). The micrograph (Figure 2) revealed a compact cellular architecture consisting of cylindrical units with walls formed by thin, layered sheets. These cylindrical blocks were tightly packed and oriented in specific directions, with only limited pore development visible at this scale. Although some authors have described coconut shell as a porous network of cylindrical tubes [67] with irregular and porous surfaces [69], the present observations underline the initially dense nature of the raw material, which will need to be structurally modified during carbonization and activation to develop a suitable porosity.

The SEM micrographs obtained for samples C-600 and C-900 (Figure 2) reveal surfaces with a high degree of roughness, mainly associated with shallow grooves, and an almost complete absence of cavities or entrances to wide porosity. This morphology indicates that carbonization alone does not generate a well-developed porous network, and that the resulting solids remain essentially compact and dense. According to Achaw, pyrolysis of coconut shell induces major transformations in the molecular structure of the material as a consequence of fusion of the cellular layers and interconnection of adjacent cell walls, which leads to collapse of the original biological architecture and limits pore accessibility. In addition, inorganic matter is clearly visible on the surface of both carbonized samples, reflecting the concentration of ash-forming species after devolatilization. A comparative inspection of the micrographs suggests that the content of disorganized or possibly extraneous material is lower in C-900 than in C-600, which can be attributed to the higher carbonization temperature promoting a more consolidated and aromatized carbon matrix.

Fourier-transform infrared (FT-IR) spectroscopy was used to identify the main functional groups in CS. The spectrum was interpreted considering that hemicellulose, cellulose, and lignin are the main biopolymers in coconut shell. As well as their chemical structures and functional groups, the assignment of the most intense absorption bands recorded in the spectrum was carried out according to the literature [69,70], as presented in Table 3. Most of the prominent absorption bands were associated with lignin-related vibrations, in agreement with literature data [70,71], and included typical O–H stretching, C–H stretching of aliphatic groups, C=O stretching of carbonyl functionalities (ketones, aldehydes, carboxylic acids and esters), C=C stretching in aromatic rings, and C–O stretching in ether and alcohol structures. These results confirm the complex oxygenated surface chemistry characteristic of lignocellulosic precursors [71].

**Figure 3:**
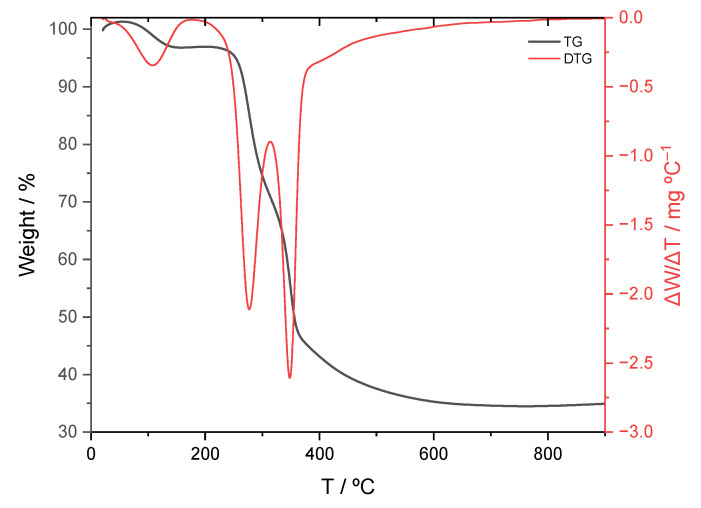
TG-DTG analyses. Sample: CS.

The TG–DTG curves of the coconut shell precursor (Figure 3) show several well-defined mass-loss regions that can be associated with the main stages of thermal decomposition of lignocellulosic materials. The initial weight loss observed below approximately 150 °C corresponds to the removal of physically adsorbed moisture and light volatile compounds. This step is accompanied by a weak DTG signal, indicating a limited mass loss.

The main decomposition region extends roughly from 200 to 380 °C and is characterized by intense DTG peaks. In this temperature interval, hemicellulose and cellulose undergo thermal degradation. Hemicellulose decomposes at lower temperatures, giving rise to the first DTG maximum, while cellulose degradation occurs at slightly higher temperatures and is responsible for the sharpest DTG peak, associated with the most pronounced mass loss. These processes account for the major reduction in sample weight and reflect the breakdown of the polysaccharide framework of the coconut shell.

At temperatures above approximately 380–400 °C, the TG curve shows a much slower and gradual mass loss, accompanied by a weak DTG signal. This region is attributed mainly to the progressive decomposition of lignin and to the rearrangement and aromatization of the remaining carbonaceous structure. Beyond about 600 °C, the mass becomes nearly constant, indicating the formation of a thermally stable carbon-rich residue. The clear separation of these thermal regions in the revised TG–DTG plot allows an unambiguous identification of the decomposition steps and provides a sound basis for selecting appropriate carbonization temperatures in the preparation of the activated carbons [72].

### 2.2. Carbonized Products

Coconut shell was subjected to carbonization under a nitrogen atmosphere at 600 °C and 900 °C for 2 h to obtain samples C-600 and C-900. The resulting yields (33.0% in weight for C-600 and 23.2% for C-900) confirmed the strong influence of the temperature of the carbonization on the mass loss of the precursor. At temperatures below 600 °C, lignocellulosic substrates undergo extensive thermal degradation [73], generating a high release of volatiles and a significant mass reduction associated with progressive aromatization of the remaining solid. At higher temperatures, such as 900 °C, the aromatic character of the material increases further, while the additional loss of mass is substantially lower.

Elemental composition analysis of C-600 and C-900 (Table 2), compared with the original precursor, revealed a decrease in hydrogen and oxygen and a simultaneous increase in carbon and nitrogen contents after pyrolysis. The ash content is slightly higher in C-600 and C-900 than in CS, which is expected since a large proportion of volatile matter is released during carbonization. The slightly higher ash content of C-600 compared to C-900 suggests that a fraction of the inorganic matter in CS is thermally unstable and is lost during pyrolysis at the higher temperature (900 °C).

The SEM micrographs obtained for C-600 and C-900 (Figure 2) suggest the presence, in C-600 and C-900, of a surface with a high degree of roughness, due to not very deep grooves, and the almost total absence of cavities and entrances to wide porosity. According to Achaw [74], pyrolysis of CS induces major transformations in the molecular structure of the material because of the fusion of cell layers and the interconnection between cell walls. The inorganic matter present on the surface of the samples is also clearly visible in the micrographs. Additionally, the content of disorganized or possibly extraneous material appears to be lower in C-900 than in C-600.

#### 2.2.1. Textural Characterization of Carbonized Products

The N_2_ adsorption isotherms at −196 °C confirmed that both carbonized products exhibit extremely low adsorption capacity, especially C-600, indicating that the development of micro- and mesoporosity is very limited. The isotherms are consistent with Type I behavior in the BDDT classification [75,76]. The textural parameters calculated from the N_2_ adsorption isotherms (Table 4) reveal that carbonized CS products are very scarcely porous solids with low surface areas. In the best case, W_0_ = 0.07 cm^3^ g^−1^ and S_BET_ = 129 m^2^ g^−1^.

Values of meso- and macroporous revealed that C-600 is predominantly meso/macroporous, whereas C-900 presents negligible meso- and macroporosity, which is compatible with sintering or shrinkage of pores during high-temperature treatment.

#### 2.2.2. Surface Chemical Characterization of Carbonized Products

**Figure 4:**
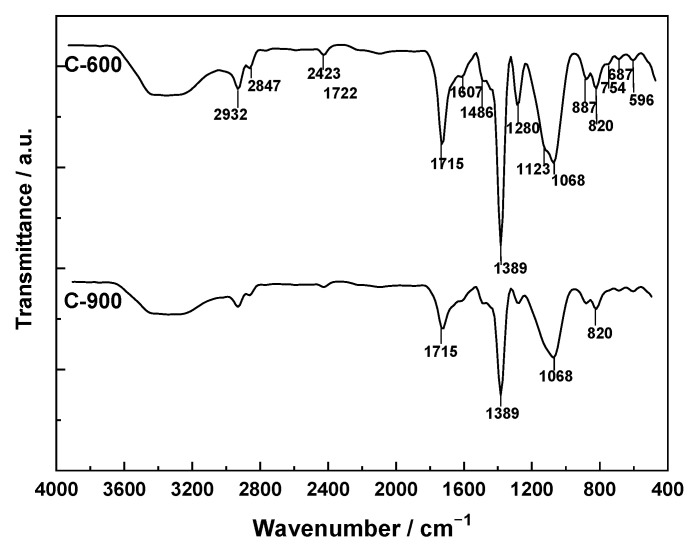
FTIR spectra of the carbonized samples.

The FT-IR spectra of C-600 and C-900 are shown in Figure 4.

The presence of a series of absorption bands located between 4000 and 450 cm^−1^ can be assigned to the bond vibrations indicated in Table 5. It can be observed that the main differences between the two spectra concern the band intensities, which are greater in the C-600 spectrum than in the C-900 spectrum, while the number of bands is practically the same in both spectra. The intensity of the absorption bands indicates that the most abundant functional groups in the carbonized products are phenolic hydroxyl groups and carboxylic acid groups, as well as ether-type structures.

### 2.3. Physically Activated Carbons

Physical activation of the carbonized intermediates was carried out using air, CO_2_, or steam, resulting in samples A-O_2_, A-CO_2_, and A-ST, respectively. It should be noted that the physical activation treatments using air, CO_2_, and steam were not conducted under identical thermal conditions. This was an intentional and necessary aspect of the experimental design, dictated by the fundamentally different oxidation strengths and activation mechanisms of the three activating agents. Air (O_2_) is a highly reactive oxidizing agent, and at elevated temperatures, it promotes rapid combustion of carbonaceous materials rather than controlled activation, leading to excessive mass loss and structural degradation. Consequently, air activation must be carried out under significantly milder conditions (lower temperature and shorter residence time) to enable limited gasification and porosity development while avoiding burning.

In contrast, CO_2_ and steam are considerably less aggressive activating agents and require higher temperatures and/or longer treatment times to effectively react with carbon and generate porosity. The activation conditions selected for each agent were therefore optimized to ensure controlled activation with reasonable yields, following established practices reported in the literature. Accordingly, the comparison among physically activated samples is not intended to isolate temperature or time as independent variables, but rather to evaluate the overall efficiency of each activation strategy under appropriate operating conditions for that specific agent.

The activation yield (Table 4) reflects that the extent of partial gasification of the carbon atoms in the carbonized products follows the order A-O_2_ > A-ST > A-CO_2_. This can be attributed to the fact that the precursors were different for A-O_2_ (C-600) and for A-CO_2_ and A-ST (C-900). Considering that the major devolatilization and mass loss of lignocellulosic precursors occurs below approximately 600 °C, carbonization at 900 °C is expected to result primarily in a higher degree of aromatization of the carbon matrix, with only a limited additional mass loss. The selection of two carbonization temperatures was therefore intended to generate carbonized intermediates with different structural features, allowing the influence of the subsequent activation treatments on porosity development and adsorption performance to be investigated, rather than to establish a strict single-variable comparison. Other factors influencing the yield include the size and geometry of the activating agent molecule. Diffusion into substrate pores becomes more difficult as molecular size increases, particularly for angular geometries. Likewise, the presence of an inert gas in the activating gas stream may influence the gasification of the carbonized material and, ultimately, the effect of activation on the porous texture of the resulting activated carbon.

Elemental analysis of the activated carbons (Table 2) indicated an increase in carbon content and a decrease in that of hydrogen and nitrogen. A-O_2_ also exhibited the highest oxygen content due to the formation of surface oxygenated groups of carbon. The ash contents of the activated samples remain very low, even compared with typical commercial activated carbons, illustrating the structural advantages of coconut-shell-derived materials for adsorption and catalytic applications.

SEM analysis (Figure 2) revealed substantial porosity development in all activated carbons and demonstrated that pore morphology depends strongly on the activating agent. SEM micrographs of A-O_2_, A-CO_2_, and A-ST clearly show that activation of the carbonized product produces porosity development that depends on the activating agent. Large cavities or pores are particularly evident in the case of A-O_2_. It is also noteworthy that pore shape depends on the activating agent: slit-shaped in A-O_2_, cylindrical in A-CO_2,_ and irregular in A-ST [77]. It can be stated that the extent of the porosity development strongly depends on the activating agent. As reported by Achaw, prolonged thermal treatment at high temperature during activation induces changes in the carbon matrix associated with softening and plastic deformation of the material, which result in erosion of pore walls, narrowing of pore widths, and transformation of initially cylindrical pores into partially flattened structures. In the present case, large cavities and wide pore openings are particularly evident in A-O_2_, reflecting the aggressive and superficial nature of air activation. Moreover, pore shape varies systematically with the activating agent: slit-shaped pores are predominant in A-O_2_, cylindrical or tubular pores are observed in A-CO_2_, and more irregular pore geometries characterize A-ST. These observations confirm that physical activation not only promotes pore formation but also determines pore morphology depending on the gasifying medium.

#### 2.3.1. Textural Characterization of Physically Activated Carbons

The textural parameters calculated from the N_2_ adsorption isotherms obtained for A-O_2_, A-CO_2_, and A-ST are summarized in Table 4. From the textural data, it is evident that the physical activation of CS carbonized products is an effective method to prepare activated carbons with a high degree of development of surface area and porosity, especially in the micropore region, although to different extents depending on the activating agent, which also affects the pore size distribution of the activated carbon samples.

The shapes of the N_2_ adsorption isotherms measured for these activated carbons resemble (Figure S1, Supplementary Materials), to a greater or lesser extent, a Type I isotherm in the BDDT classification system, and therefore it can be asserted that they are essentially microporous solids, as is further supported by the values of S_BET_, W_0_, and V_mi_. The variation in the amount adsorbed with increasing relative equilibrium pressure (p/p_0_) indicates that the pore size distribution in the micropore domain becomes broader in the order A-ST > A-CO_2_ > A-O_2_. In the mesopore region, pore size distribution is quite similar for the three activated carbon samples.

In addition, S_BET_, W_0_, or V_mi_, and V_me_ vary according to the following order: A-ST > A-CO_2_ >> A-O_2_. S_BET_ reaches 738 m^2^ g^−1^ for A-ST, which contrasts sharply with the value of 270 m^2^ g^−1^ obtained for A-O_2_. The slight increase in the amount adsorbed with p/p_0_ suggests that these carbons not only contain a large volume of micropores, but also mesopores of different sizes. The mesopore volume (V_me_) has similar values for the three activated carbons, ranging from 0.06 cm^3^ g^−1^ for A-O_2_ to 0.08 cm^3^ g^−1^ for A-CO_2_. In any case, it should be emphasized that these mesopore volumes are relatively low compared with other activated carbons, whether synthetic or commercial.

Mercury intrusion curves for A-O_2_, A-CO_2_, and A-ST (Figure S2, Supplementary Materials) show, first, that the pore size distribution in the macropore region is very similar for A-O_2_ and A-CO_2_. In the mesopore region, the presence of a fraction of narrow mesopores is noteworthy only in the case of A-CO_2_. By contrast, A-ST exhibits a very different pore size distribution, containing narrow macropores and wide mesopores. Thus, activation in air, CO_2_, or steam makes it possible to prepare activated carbons with pores of different sizes in the meso- and macropore regions. In any case, it should be noted that porosity development in both regions is limited, particularly when steam is used as the activating agent. The macropore volume V_ma-p_ reaches, at most, 0.11 cm^3^ g^−1^ in A-O_2_. When air is used as an activating agent, the generation of wide pores, such as macropores, is generally associated with a rapid gasification process. This process makes the activation effect more superficial than deep, and dilutes O_2_ in the gas stream with N_2_. In relative terms, V_ma-p_ and V_me-p_ are appreciable only for A-CO_2_, whereas these pore volumes are very close to zero in A-ST.

#### 2.3.2. Surface Chemical Characterization of Physically Activated Carbons

**Figure 5:**
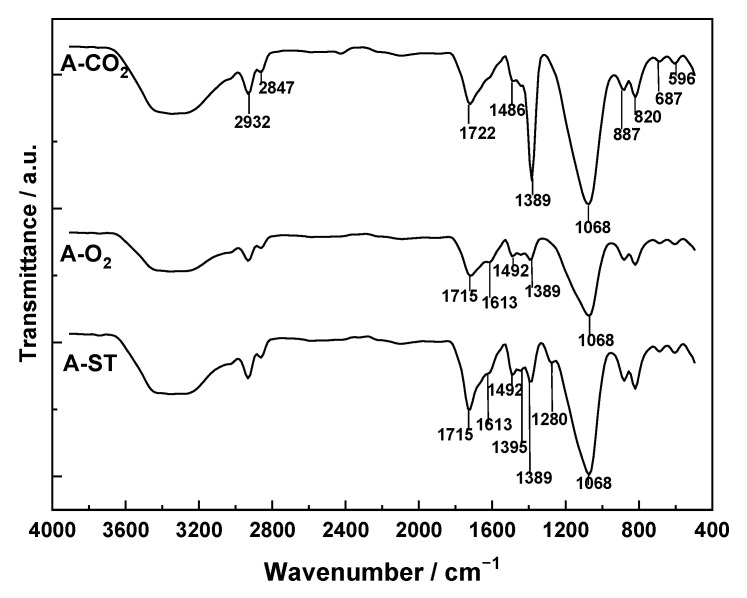
FT-IR spectrum. Samples: A-O_2_, A-CO_2_, and A-ST.

The FT-IR spectra recorded for A-O_2_, A-CO_2_, and A-ST (Figure 5) exhibit essentially the same series of absorption bands, although with different intensities. The presence of the same functional groups on the surface of the three activated carbons is consistent with the fact that activated carbon was always prepared through an oxidation process of the carbonized products; consequently, there is a general tendency towards an increase in the oxidation state of the carbon via the formation of oxygenated structures. However, the extent to which each organic functional group is formed depends more on kinetic factors than on thermodynamic ones. Thus, the progress of oxidation with each of the three oxidizing agents during the activation treatment time will be the determining factor for the concentration of the groups finally present on the carbon surface. In the extreme case, complete mineralization of the carbonized product could even occur. The values of the activation yield (Table 4) provide an idea of the degree of conversion of the carbonized materials.

The most intense bands present in the spectra can be assigned to the following bond vibration modes: 3400 cm^−1^, ν(O–H); 2932 and 2847 cm^−1^, ν(C–H); 1722 cm^−1^, ν(C=O); 1613 and 1492 cm^−1^, skeletal ν(C–C); 1389 cm^−1^, δ(C–H); 1068 cm^−1^, ν(C–O). As an example, it may be noted that the intensity of the band centered at 1722 cm^−1^ is stronger in the order A-ST > A-CO_2_ > A-O_2_, which reflects the relative abundance of C=O-containing groups in the samples. It is also noteworthy that the band with a maximum at 1060 cm^−1^ has the same intensity in the spectra of A-CO_2_ and A-ST and is much weaker in the spectrum of A-O_2_.

As can be seen in Table 4, the pH_pzc_ is above 7.0 for A-CO_2_ and A-ST, and slightly below 7.0 for A-O_2_. The basic character of the former two carbons over such a broad pH range may be attributed to the presence of C=O groups in pyrone-type structures, π-electrons in graphene layers, heteroatoms such as nitrogen, and inorganic impurities, among others. When interpreting pH_pzc_ values, it must also be considered that this pH arises from the balance between acidic and basic groups on the surface of each carbon and that, according to the volumetric titration method used, it depends not only on the relative contents of these groups but also on their acid–base strength. It should be noted that A-CO_2_ and A-ST, which have the highest pH_pzc_ values, also possess the highest content of C–O-containing surface structures.

### 2.4. Chemically Activated Carbons

Chemical activation of coconut shell was carried out using ZnCl_2_, H_3_PO_4_, and KOH as activating agents, resulting in samples A-ZnCl_2_, A-H_3_PO_4_, and A-KOH, respectively. It should be emphasized that the activation temperature used for the preparation of A-KOH was higher than that employed for the other chemically activated carbons, and this choice was dictated by the intrinsic activation mechanism of KOH. Unlike ZnCl_2_ or H_3_PO_4_, KOH requires elevated temperatures to effectively promote carbon activation through a sequence of redox reactions, intercalation of potassium species into the carbon matrix, and subsequent gasification processes that lead to extensive pore development. At lower temperatures, KOH would mainly act as a dehydrating or surface-modifying agent, resulting in insufficient activation.

Therefore, the activation conditions were selected to ensure that each chemical agent operated within the temperature range necessary for its effective function as an activator, rather than to impose identical thermal histories across all samples. The comparison among chemically activated carbons is thus intended to assess the overall performance of different activation strategies under appropriate and realistic conditions for each activating agent. Differences in adsorption capacity should consequently be interpreted as the combined outcome of activation mechanism, pore development, and surface chemistry, rather than being attributed solely to the activating agent or to temperature as an isolated variable.

The values of R_I_, R_A_, and R_P_ calculated for the activated carbons prepared by chemical activation with ZnCl_2_, H_3_PO_4_, and KOH show that R_I_ varies according to the sequence A-H_3_PO_4_ > A-ZnCl_2_ > A-KOH (Table 6).

Moreover, R_I_ is very high for all three activating agents, particularly for H_3_PO_4_. Thus, it is evident that when the aqueous H_3_PO_4_ solution is brought into contact with the lignocellulosic substrate during the impregnation step, most of the acid present in the medium is retained by the material [78]. In the case of H_3_PO_4_, retention by the lignocellulosic support is probably facilitated and enhanced by the strong tendency of this substance—depending on concentration—to undergo polymerization. According to the literature, two or more H_3_PO_4_ molecules condense with the elimination of water molecules to form polyphosphoric acid, which is a complex mixture of linear molecules differing in chain length.

The fact that R_A_ is much lower than R_I_ is due to the release of volatile matter when the impregnated products are subjected to high-temperature treatment during the preparation of the activated carbon samples. Volatile matter is likely generated both by the pyrolysis of the parts of the lignocellulosic substrate that were not impregnated—and, thus, not chemically altered by the activating agent during impregnation—and by the chemical interaction between the activating agent and the substrate. The particularly low value of R_A_ for A-KOH compared with A-H_3_PO_4_ and A-ZnCl_2_ is consistent with the higher carbonization temperature used (750 °C instead of 500 °C) for the product impregnated with this activating agent.

Based on the values of R_P_, and hence the overall mass balance, the chemical activation method that appears most advantageous from a practical standpoint is that using ZnCl_2_ as activating agent, followed by H_3_PO_4_. In contrast, the least favorable method is chemical activation with KOH. The very low yield obtained with this agent (R_P_ = 3.04%) is related to the high chemical reactivity of elemental potassium and potassium compounds in general, particularly at high temperatures. This reactivity may be enhanced by increased impregnation of the lignocellulosic substrate with molten KOH (melting point 360 °C) during the carbonization/activation stage in the preparation of A-KOH.

Elemental and proximate analysis (Table 2) revealed significant compositional differences among the chemically activated carbons. All samples displayed high carbon content, although the values followed the sequence A-ZnCl_2_ > A-H_3_PO_4_ > A-KOH. A-ZnCl_2_ exhibited the lowest ash content, a particularly advantageous feature given that inorganic matter strongly influences the applicability of activated carbon in adsorption and catalysis. The higher oxygen percentage detected in A-H_3_PO_4_ and A-KOH reflects the presence of oxygenated functional groups generated during activation and/or incorporated from residual species.

SEM micrographs of the chemically activated samples A-ZnCl_2_, A-H_3_PO_4_, and A-KOH (Figure 2) further demonstrate the strong influence of the activating agent on surface morphology. The surface of A-ZnCl_2_ appears highly irregular, with clearly visible entrances to cylindrical pores, a feature that bears some resemblance to that observed for A-CO_2_. In contrast, A-H_3_PO_4_ exhibits a comparatively smooth surface, dominated by large cavities that can act as access points to the internal porosity, consistent with the pore-opening and swelling effects associated with phosphoric acid activation. Finally, A-KOH displays a distinctive tessellated surface pattern, with a high degree of regularity over most of the imaged area, except for the central region. Although the origin of this morphology is not straightforward, it is likely related to the intense chemical reactivity of KOH at high temperature, which promotes extensive restructuring of the carbon framework during activation.

The N_2_ adsorption isotherms measured for A-ZnCl_2_, A-H_3_PO_4_, and A-KOH (Figure S3, Supplementary Materials) show that the isotherms corresponding to A-ZnCl_2_ and A-KOH are quite like a Type I isotherm in the BDDT classification, whereas the isotherm of A-H_3_PO_4_ more closely resembles a Type IV isotherm. The latter may also be interpreted as a composite isotherm combining Type I and Type IV features. The shape of the isotherms (in particular the width of the knee and the evolution of N_2_ adsorption with increasing p/p_0_) is consistent with the presence of almost exclusively narrow micropores in A-ZnCl_2_, a broad pore size distribution in the micropore region and also in the mesopore region—but only for relatively narrow mesopores—of different sizes in A-KOH, and a very broad pore size distribution in both the micro- and mesopore regions in A-H_3_PO_4_. In summary, the N_2_ adsorption isotherms indicate that the sample with the most heterogeneous porosity in the micro- and mesopore regions is A-H_3_PO_4_.

The absence, over the whole p/p_0_ range, of a clear inflection point in the isotherm of A-H_3_PO_4_, indicative of the onset of capillary condensation, suggests that the mesopores present in this sample are wide enough for adsorption to proceed only via the usual monolayer–multilayer mechanism. The relative position of the isotherms with respect to the *y*-axis indicates that microporosity development varies according to the order A-KOH > A-H_3_PO_4_ > A-ZnCl_2_, while for mesoporosity the order is A-H_3_PO_4_ > A-KOH > A-ZnCl_2_. As shown in Table 4, S_BET_ ranges from 992 m^2^ g^−1^ for A-ZnCl_2_ to 1798 m^2^ g^−1^ for A-H_3_PO_4_. W_0_ is 0.53 cm^3^ g^−1^ for A-ZnCl_2_ and 0.70 cm^3^ g^−1^ for A-KOH, while V_me_ is 0.03 cm^3^ g^−1^ for A-ZnCl_2_ and 0.61 cm^3^ g^−1^ for A-H_3_PO_4_.

The cumulative pore volume versus pore radius curves obtained for A-ZnCl_2_, A-H_3_PO_4_, and A-KOH (Figure S4, Supplementary Materials) show that meso- and macroporosity are very poorly developed in A-ZnCl_2_; that the porosity of A-H_3_PO_4_ consists of macropores and, above all, mesopores of different sizes; and that A-KOH exhibits rather wide macropores with pore radii greater than approximately 3000 Å. It should be noted that, in the latter case, the porosity measured by mercury porosimetry (Table 4) may result from the packing or agglomeration of carbon particles, due to the high degree of activation achieved in its preparation, rather than from true penetration of mercury into carbon pores under high pressure during the porosimetry experiment. Consistent with these observations, mesopore volume V_me-p_ reaches 0.44 cm^3^ g^−1^ for A-H_3_PO_4_, whereas macropore volume V_ma-p_ reaches 0.85 cm^3^ g^−1^ for A-KOH; for this carbon, V_me-p_ is essentially zero. These results clearly show that, starting from CS and using chemical activation with ZnCl_2_, H_3_PO_4_, and KOH, it is possible to obtain activated carbons with markedly different meso- and macroporous structures: weakly meso-/macroporous with ZnCl_2_, both meso- and macroporous with H_3_PO_4_, and predominantly macroporous with KOH. High specific surface areas obtained by chemical activation using H_3_PO_4_ or KOH are not exclusive to coconut shell and have also been reported for activated carbons derived from other agricultural and biomass precursors. For instance, phosphorus-containing activated carbons prepared from honeydew using H_3_PO_4_ activation exhibit extensive microporosity and high surface areas as a result of dehydration, crosslinking, and pore-forming reactions promoted by phosphorus species [79]. Similarly, KOH-activated porous carbons derived from hemp-based precursors show highly developed micro- and mesoporous structures, attributed to the well-known redox and intercalation mechanisms associated with KOH activation at high temperature [80]. In this context, the coconut-shell-derived activated carbons prepared in the present work display textural properties that are fully comparable to those reported for other agricultural precursors, while offering the additional advantages of high fixed carbon content, structural rigidity, and widespread availability of coconut shell waste.

**Figure 6:**
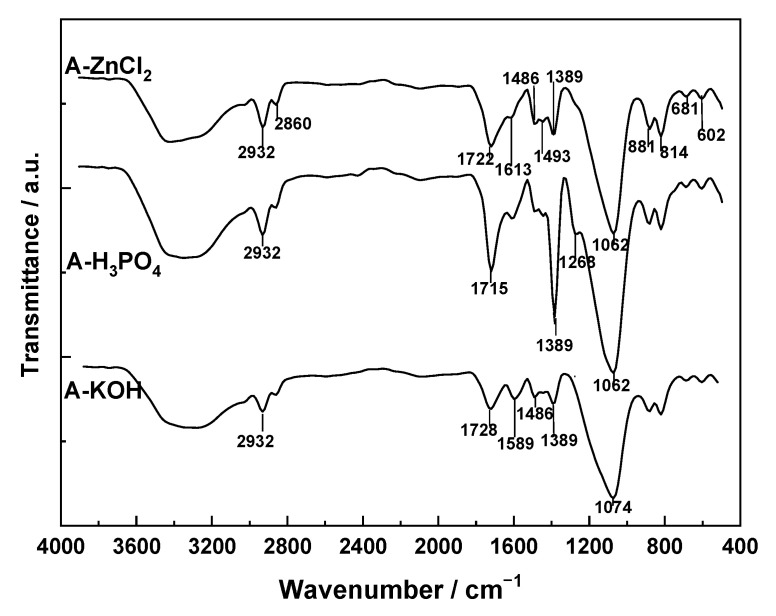
FT-IR spectrum. Samples: A-ZnCl_2_, A-H_3_PO_4_, and A-KOH.

The FT-IR spectra recorded for A-ZnCl_2_, A-H_3_PO_4_, and A-KOH (Figure 6) are very similar to one another. In all spectra, the most intense absorption bands show maxima around 3400, 1720, and 1070 cm^−1^, which can be assigned to ν(O–H) vibrations of phenolic hydroxyl groups, ν(C=O) of carboxylic acid groups, and ν(C–O) of ether-type structures, respectively. Other, less intense bands with maxima around 2830, 2850, and 1380 cm^−1^ are likely due to C–H vibrations in methyl and methylene groups.

Since all the samples were prepared in the 400–900 °C range for carbonization and/or activation, a large fraction of the surface functional groups is thermally unstable. Then, thermal decomposition for carboxylic and lactonic groups occurs between 200 and 700–800 °C with CO_2_; for quinone, phenolic and ether groups between 500 and 1000 °C with CO emission; for phenolic groups between 200–300 °C and 400–500 °C with H_2_O formation and from hydrogen bound to C and O between 500 and 700 °C with release of H_2_ formed [81].

Measurement of the pH at the point of zero charge (Table 4) confirmed that the surface of the chemically activated carbons is acidic, following the order of acid strength A-H_3_PO_4_ > A-ZnCl_2_ > A-KOH. The very low value of pH_pzc_ = 2.5 for A-H_3_PO_4_ is particularly remarkable. It is noteworthy that there is a good correlation between pH_pzc_ and the intensity of the absorption band at 1715–1728 cm^−1^ in the FT-IR spectra, which has been assigned to ν(C=O) vibrations in carboxylic acid groups. The variation of pH_pzc_ must be considered when interpreting adsorption results for organic or inorganic solutes in solutions, since pH_pzc_ indicates the pH at which the surface charge of the carbon changes sign from positive to negative, which may influence the adsorption mechanism. Moreover, these results provide valuable information on the pH range over which adsorption of a given solute will be more favorable, depending on whether the solute is cationic or anionic.

### 2.5. Comparative Textural and Chemical Characterization of All Samples

The comparison with respect to the textural characterization (Table 4) demonstrates that (i) carbonization alone produces insignificant porosity, (ii) physical activation increases microporosity progressively depending on the activating agent (air < CO_2_ < steam), and (iii) chemical activation—which introduces chemical templating and intense gasification—produces the most advanced textural development, especially in the cases of H_3_PO_4_ and KOH. These differences justify combining carbonization with activation to tailor pore size distribution for adsorption applications involving molecules of different sizes. The high specific surface area of the activated carbons is mainly associated with the development of microporosity, i.e., pores with diameters smaller than 2 nm. Because micropores provide a very large internal surface per unit mass, their presence and volume play a dominant role in determining the BET surface area, whereas wider mesopores contribute primarily to pore accessibility and mass transport rather than to surface area itself.

With respect to the chemical characterization, in summary, carbonization reduces oxygen functional groups and enhances aromaticity; physical activation reintroduces oxygen to varying degrees depending on the activating agent, and chemical activation produces more diverse and highly reactive surface chemistries, ranging from strongly acidic (H_3_PO_4_) to weakly acidic (KOH). These differences rationalize the use of multiple activation strategies depending on the target adsorbent–adsorbate interactions: acidic carbons are expected to favor adsorption of basic species, whereas basic carbons facilitate adsorption of acidic or anionic contaminants.

The FT-IR spectra of A-O_2_, A-CO_2_, and A-ST exhibit essentially the same set of characteristic absorption bands, indicating the presence of similar oxygen-containing surface functionalities on the three physically activated carbons. The dominant bands correspond to ν(O–H) (≈3400 cm^−1^), ν(C=O) (≈1720 cm^−1^), and ν(C–O) (≈1070 cm^−1^), associated with phenolic, carboxylic, and ether-type structures, respectively. However, the relative intensity of these bands varies systematically with the activating agent. The ν(C=O) band at ≈1720 cm^−1^ shows the order A-ST > A-CO_2_ > A-O_2_, indicating a higher abundance of carbonyl-containing groups on A-ST and A-CO_2_, whereas A-O_2_ exhibits the weakest development of oxygenated functionalities. A similar trend is observed for the ν(C–O) band around 1060 cm^−1^, which is significantly less intense in A-O_2_ than in A-CO_2_ and A-ST.

These differences in surface chemistry are consistent with the measured pH_pzc_ values. A-CO_2_ and A-ST display basic behavior (pH_pzc_ > 7), which can be attributed to their higher content of pyrone-type C=O structures, π-electron density in graphene layers, and possible contributions from heteroatoms. Conversely, A-O_2_ presents a slightly acidic character (pH_pzc_ < 7), reflecting a higher proportion of acidic surface groups relative to basic ones. Since pH_pzc_ reflects the balance between acidic and basic functionalities, the agreement between FT-IR trends and pH_pzc_ values confirms that activation with CO_2_ or steam, unlike activation in air, promotes the formation of basic oxygenated structures rather than acidic ones.

In summary, while physically activated carbons share the same qualitative functional groups, the degree of surface oxidation and the acid–base character of the surface strongly depend on the activating agent, following the sequence: surface oxygenation: A-ST ≳ A-CO_2_ >> A-O_2_; and basicity (pH_pzc_): A-CO_2_ ≈ A-ST > A-O_2_.

The FT-IR spectra of A-ZnCl_2_, A-H_3_PO_4_, and A-KOH are highly similar, and also comparable in spectral profile to those of the physically activated carbons. All chemically activated samples exhibit the characteristic absorption bands of oxygenated functional groups: ν(O–H) ≈ 3400 cm^−1^ (phenolic hydroxyls), ν(C=O) ≈ 1720 cm^−1^ (carboxylic acids/lactones), and ν(C–O) ≈ 1070 cm^−1^ (ether-type structures). Less intense bands assigned to ν(C–H) vibrations from aliphatic –CH_3_ and –CH_2_– groups are also observable. The spectral resemblance across the three samples suggests that thermal activation at high temperature destabilizes most surface functionalities, and that partial reoxidation of the carbon surface during storage leads to the regeneration of similar oxygenated groups irrespective of the activating chemical used.

Despite the similarity in FT-IR spectra, the pH_pzc_ values indicate clear differences in the acid–base character of the three carbons. All chemically activated samples exhibit acidic behavior (pH_pzc_ < 6), with A-H_3_PO_4_ showing the lowest value (≈2.5), reflecting a strongly acidic surface enriched in oxygenated groups capable of donating protons. A-ZnCl_2_ and A-KOH show slightly higher pH_pzc_ values, although both remain in the acidic domain, indicating that their final surfaces contain a higher proportion of acidic functionalities than basic sites. This trend is in line with the oxidative character of the activating treatments and with the partial regeneration of carboxylic, phenolic, and lactonic groups upon exposure to air after synthesis.

Overall, whereas FT-IR spectra do not allow a clear discrimination among the chemically activated carbons, pH_pzc_ provides an effective criterion to differentiate their surface chemistry, highlighting the predominance of acidic oxygenated groups in all cases, with the maximum acidity observed for A-H_3_PO_4_ (surface acidity based on pH_pzc_: A-H_3_PO_4_ << A-ZnCl_2_ ≈ A-KOH).

### 2.6. Adsorption of Dyes

The main objective is to investigate the possible influence of the textural properties of activated carbon prepared from CS on the adsorption of the dyes methylene blue (MB), methyl orange (MO), and orange G (OG) in aqueous solution. These dyes were selected mainly because of the differences in their chemical composition and structure. Knowledge of the structure and geometry of the dyes is considered essential to explain their behavior as adsorbates in the adsorption process. For example, OG is a chemical species with a greater height and, above all, a greater width than MB and MO [82], dimensions that typically hinder its entry into narrow pores.

Another important factor in studies on the adsorption of dyes in aqueous solution is whether they are acidic, basic, or neutral, since in the electrostatic interactions that may occur with the adsorbent, it is necessary to consider not only the sign and magnitude of the charge, but also the number of ionizable groups present in the dye ion. In our case, the charge is positive in methylene blue and negative in methyl orange and orange G (Figure S5, Supplementary Materials). Moreover, there is only one ionizable group in methylene blue and methyl orange, while orange G contains two ionizable groups. The partial charge on each of the oxygen atoms of the –SO_3_^−^ groups is equal to 2/3 of the total charge.

The data from the adsorption kinetics and the most representative isotherms are presented graphically and in tabular form in the Supplementary Materials (Tables S2–S7 and Figures S6–S9). Following the same approach as in the previous sections, the study will be carried out separately for the three groups of adsorbents, namely the carbonized products, the activated carbons prepared by the physical activation method, and the activated carbons obtained by the chemical activation method, as well as for the three dyes in the order MB, MO, and OG. In the study of the adsorption process, the kinetics will be addressed first, followed by the equilibrium (isotherms).

The kinetic and equilibrium adsorption results reveal the decisive role of the physicochemical characteristics of the adsorbents in governing dye uptake. For the carbonized products, adsorption proceeds significantly faster on C-600 than on C-900 for methylene blue (MB) and methyl orange (MO), whereas orange G (OG) displays slow uptake on both samples. This behavior is consistent with the limited accessibility of the narrow micropores that dominate C-900, which impose stronger intraparticle diffusion resistance, while the slightly wider pores of C-600 reduce steric hindrance and facilitate transport. At equilibrium, all three dyes exhibit appreciable adsorption only at high C_e_/C_0_ values, indicating weak affinity for the carbonized materials and a multilayer adsorption mechanism driven mainly by lateral adsorbate–adsorbate interactions. Among the dyes, MB consistently shows the highest capacity, suggesting that π–π stacking interactions between its aromatic rings and the basal graphene-like domains of the carbonaceous matrix can partially compensate for the poor textural properties of the carbonized samples. These trends are quantitatively reflected in the kinetic and isotherm datasets provided in the Supplementary Materials (Tables S2–S7).

The kinetics of adsorption processes are commonly analyzed using empirical rate models that allow quantitative comparison of adsorption rates and provide insight into the controlling mechanisms. Among them, the pseudo-first-order (PFO) and pseudo-second-order (PSO) kinetic models are the most widely applied to describe the uptake of organic dyes from aqueous solutions by carbonaceous adsorbents.

The pseudo-first-order model, originally proposed by Lagergren, assumes that the rate of occupation of adsorption sites is proportional to the number of unoccupied sites. It is generally expressed in its differential form as follows:

(1) $$\frac{dq_{t}}{dq_{e}}=k_{1}(q_{e}-q_{t})$$

where *q_t_* (mg g^−1^) is the amount of dye adsorbed at time *t*, *q_e_* (mg g^−1^) is the adsorption capacity at equilibrium, and *k*_1_ (min^−1^) is the pseudo-first-order rate constant. Integration with the appropriate boundary conditions leads to the linearized form:

(2) $$ln\left(q_{e}-q_{t}\right)=\ln q_{e}-k_{1}t$$

This model is often associated with physisorption processes and is particularly suitable for describing adsorption at the initial stages, when external mass transfer or diffusion through the liquid film surrounding the adsorbent particles may dominate. However, in many dye adsorption systems, the PFO model fails to accurately predict the equilibrium adsorption capacity, especially over the entire adsorption time range.

The pseudo-second-order model, developed by Ho and McKay, is based on the assumption that the adsorption rate is proportional to the square of the number of unoccupied sites and is commonly linked to chemisorption involving valence forces, electron sharing, or exchange between adsorbent and adsorbate. Its differential form is given by the following:

(3) $$\frac{dq_{t}}{dq_{e}}=k_{2}(q_{e}-q_{t}{)}^{2}$$

where *k*_2_ (g mg^−1^ min^−1^) is the pseudo-second-order rate constant. Integration yields the linear expression as follows:

(4) $$\frac{t}{q_{t}}=\frac{1}{q_{e}^{2}k_{2}}+\frac{t}{q_{e}}$$

According to the values summarized in Tables S2, S4 and S6 (Supplementary Materials), the PSO model provides an excellent fit for dye adsorption onto the carbonized samples over the full contact time range, yielding calculated *q_e_* values in good agreement with experimental data. This behavior suggests that the overall adsorption rate is controlled by surface interactions rather than by mass transfer alone. Consequently, comparison of PFO and PSO fits is widely used to identify the dominant kinetic regime and to assess the relative importance of surface chemistry and pore accessibility in adsorption systems involving organic dyes.

When moving to the physically activated carbons, the kinetic performance improves markedly. MB adsorption takes place rapidly on all samples, especially on A-CO_2_ and A-ST, in line with their large S_BET_ values and well-connected micro–mesopore networks, which minimize mass-transfer limitations and allow rapid access to internal active sites. In contrast, MO and OG display slower kinetics, highlighting the contribution of molecular size, charge distribution, and hydration radius to diffusion barriers.

The equilibrium isotherms reinforce these observations, since A-CO_2_ and A-ST consistently reach the highest adsorption capacities, while A-O_2_ performs significantly worse. The shape of the isotherms—characterized by a long plateau before the steep final rise—indicates monolayer formation followed by multilayer stacking at high C_e_/C_0_. Notably, MB is again the most strongly adsorbed dye, confirming that large aromatic cations experience favorable π–π interactions and benefit more from the availability of widened micropores. These findings are fully supported by the detailed kinetic and isotherm data in the Supplementary Materials (Tables S2–S7 and Figure S6), where the rapid decrease in C(t) for MB and the markedly higher equilibrium capacities of A-CO_2_ and A-ST can be observed.

Further improvements in adsorption performance are observed for the chemically activated carbons. A-KOH and A-H_3_PO_4_ show the fastest kinetics for all dyes, consistent with the broader micropore size distributions generated by their respective activation mechanisms, which enhance diffusion and reduce steric restrictions. In contrast, A-ZnCl_2_ exhibits substantially slower uptake, reflecting the predominance of narrower pores that impede the access of bulky aromatic molecules. At equilibrium, A-KOH displays the highest capacities, followed closely by A-H_3_PO_4_ and, by a considerable margin, A-ZnCl_2_. The superiority of A-KOH and A-H_3_PO_4_ correlates with their higher S_BET_ values and more heterogeneous pore size distributions, particularly within the micropore region. Across all chemically activated samples, MB again stands out as the most efficiently adsorbed dye, demonstrating that electrostatic attraction and π–π stacking outweigh pure size-exclusion effects. These conclusions are in agreement with the kinetic and equilibrium results summarized in the Supplementary Materials (Tables S2–S7 and Figure S7), where the steeper C(t) decay and the higher maximum dye uptake for A-KOH and A-H_3_PO_4_ are quantitatively evident.

Adsorption equilibrium data are commonly interpreted using isotherm models that describe how adsorbate molecules distribute between the liquid phase and the solid surface at constant temperature. Among the various models proposed, the Langmuir and the Freundlich isotherms are the most frequently employed to analyze the adsorption of organic dyes from aqueous solution onto activated carbons.

The Langmuir isotherm is based on a simplified theoretical framework that assumes adsorption occurs at specific, energetically equivalent sites on a homogeneous surface, leading to the formation of a monolayer of adsorbate molecules. Once a site is occupied, no further adsorption can occur at that location, and interactions between adsorbed species are neglected. The Langmuir equation is expressed as follows:

(5) $$q_{e}=\frac{q_{max}bC_{e}}{1+bC_{e}}$$

where *q_e_* (mg g^−1^) is the amount adsorbed at equilibrium, *Ce* (mg L^−1^) is the equilibrium concentration of the adsorbate in solution, *q*_*m*_ (mg g^−1^) represents the maximum monolayer adsorption capacity, and *K*_*L*_ (L mg^−1^) is the Langmuir affinity constant related to the free energy of adsorption. A good fit to the Langmuir model indicates monolayer coverage on a relatively uniform surface and suggests that adsorption sites are finite and equivalent. In dye adsorption studies, the Langmuir model is often used to estimate the maximum uptake capacity of activated carbons.

In contrast, the Freundlich isotherm is an empirical model that accounts for adsorption on heterogeneous surfaces with a non-uniform distribution of adsorption energies. It assumes that stronger binding sites are occupied first and that adsorption capacity increases continuously with concentration. The Freundlich equation is given by the following:

(6) qe=KFCe1n

where *K*_*F*_ ((mg g^−1^)(L mg^−1^)^1/*n*^) is the Freundlich constant related to adsorption capacity, and *n* is a heterogeneity factor that reflects the intensity of adsorption. Values of 1/*n* between 0 and 1 indicate favorable adsorption and increasing surface heterogeneity.

The adsorption equilibrium of MB, MO, and OG on the carbonized samples is generally weak and poorly defined, reflecting the very limited porosity and surface development of these materials. For MB adsorption, C-600 shows a modest fit to the Freundlich model (R^2^ = 0.902, Table S3), whereas the Langmuir model provides a poorer description (R^2^ = 0.760), indicating heterogeneous adsorption on a low-energy surface with no clear monolayer saturation. In contrast, C-900 exhibits a better Langmuir fit for MB (R^2^ = 0.947), suggesting a more uniform adsorption behavior, although the estimated monolayer capacity (Q_0_ ≈ 0.26 × 10^−3^ mol g^−1^) remains low.

For MO and OG, the equilibrium data for C-600 cannot be satisfactorily described by the Langmuir model (Tables S5 and S7), and the Freundlich parameters indicate unfavorable adsorption, consistent with steric limitations and poor accessibility of adsorption sites. Although C-900 shows acceptable Langmuir fits for MO and OG, the associated capacities and affinity constants remain small, confirming that carbonization alone does not produce adsorbents suitable for effective dye removal.

In contrast, the activated samples exhibit well-defined adsorption equilibria, with significantly improved fits to both Langmuir and Freundlich models. For MB adsorption, physically activated carbons (A-CO_2_ and A-ST) show excellent Langmuir correlations (R^2^ ≥ 0.987), indicating monolayer-dominated adsorption on energetically uniform sites, while A-KOH displays the highest monolayer capacity (Q_0_ ≈ 2.14 × 10^−3^ mol g^−1^), consistent with its highly developed microporosity. Freundlich exponents (1/n < 0.5) further confirm favorable adsorption on heterogeneous surfaces.

For MO and OG, Langmuir fits are particularly good for A-CO_2_, A-ST, and A-ZnCl_2_ (R^2^ ≈ 0.99), indicating that despite the larger molecular size and anionic character of these dyes, adsorption proceeds predominantly via site-limited mechanisms when sufficient pore accessibility is provided. A-H_3_PO_4_ shows strong Freundlich behavior, reflecting the contribution of mesoporosity and surface heterogeneity. Overall, the equilibrium analysis confirms that activation—especially with steam, CO_2_, and KOH—transforms coconut shell into high-performance adsorbents, where pore structure and surface chemistry jointly govern dye uptake.

Taken together, these results provide coherent evidence that adsorption performance results from the complex interplay of textural development and surface chemistry. Broad microporosity, enhanced micro–mesopore connectivity, and high S_BET_ are the main drivers of fast intrapore diffusion and high equilibrium uptake, while favorable aromatic interactions and surface charge effects provide an additional level of selectivity, particularly for MB. The integration of the supplementary kinetic and isotherm datasets with the comparative discussion confirms that tailoring pore size distribution remains the most influential design variable for optimizing the adsorption of organic dyes in aqueous solution.

#### 2.6.1. Comparison of Results

**Figure 7:**
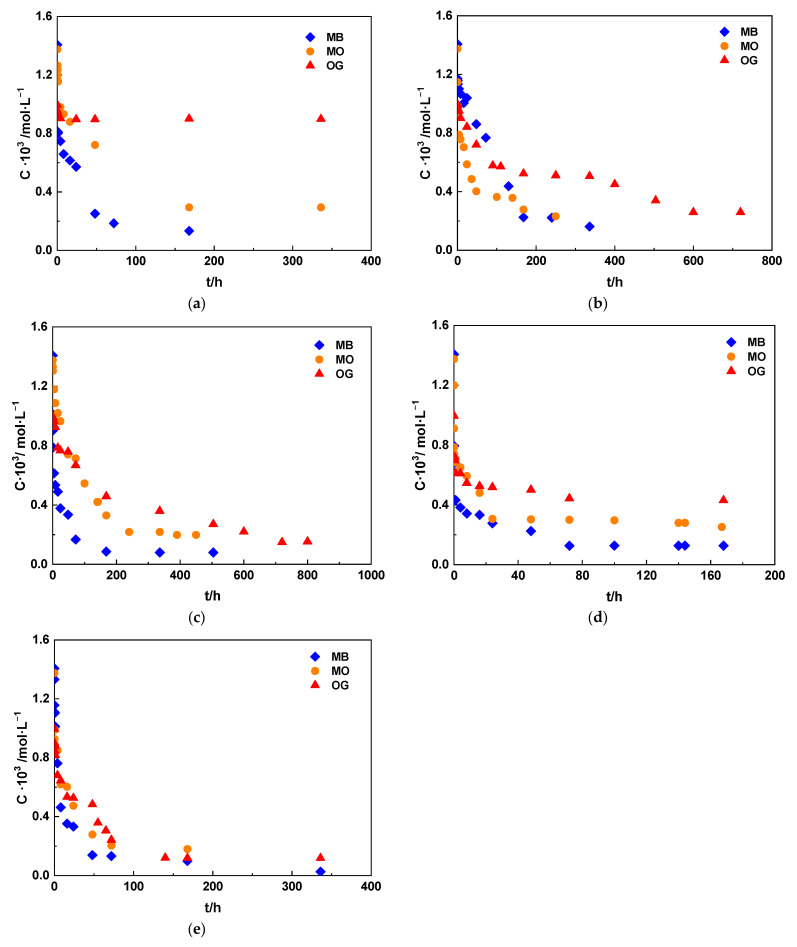
Adsorption of MB, MO, and OG. Kinetics. Sample: (**a**) C-900; (**b**) A-ZnCl_2_; (**c**) A-ST; (**d**) A-KOH; (**e**) A-H_3_PO_4_.

The results obtained in the present work, which have been presented, described, and discussed in this section, clearly demonstrate the strong influence of the preparation method of the carbonaceous adsorbents on the kinetics and equilibrium of the adsorption process of the dyes in aqueous solution. In this study, an attempt has been made to establish a correlation between the adsorption results and the textural properties of the adsorbents prepared by the same carbonization or activation method, although applying different heating conditions and using different activating agents, and the size and geometry of the adsorbate ions. However, on a comparative basis, the adsorption behavior of the different dyes was not examined as a function of specific textural properties of the adsorbent, which are usually relevant in relation to the adsorption process. Consequently, and as a way of completing the information on the adsorption process, it has been considered interesting to compare, as is performed below, the kinetic adsorption results of the three dyes using carbonaceous adsorbents texturally characterized as possessing, in particular, porosity of difficult access (incipient, blocked, constricted, etc.; C-900) or narrow micropores (A-ZnCl_2_), wide micropores (A-ST, A-KOH) and mesopores of different sizes (A-H_3_PO_4_). For clarity, it should be noted that samples A-O_2_ and A-CO_2_ are not included in the comparative kinetic plots shown in Figure 7. These samples exhibited relatively low and poorly differentiated adsorption performance, which was not representative of the adsorption regimes discussed in this section. Figure 7 was therefore constructed using selected materials that illustrate distinct textural situations—incipient or poorly accessible porosity, narrow microporosity, wide microporosity, and significant mesoporosity—in order to better highlight the influence of pore structure on dye adsorption kinetics. Inclusion of A-O_2_ and A-CO_2_ would not have provided additional mechanistic insight and would have reduced the clarity of the comparison.

This figure clearly shows the determining influence of the porous texture of the adsorbent on the kinetics of the adsorption process of dyes in aqueous solution. As inferred, in general, the ease and extent of dye adsorption follow the order MB > MO > OG, which is not the expected one based on the ionic size of the adsorbates: MB > OG > MO (OG and MO differ more noticeably in ion width). With regard to the carbonaceous adsorbents, the clearest example of adsorption behavior can be obtained from the figure. For C-900, which most likely has incipient porosity that is poorly developed and perhaps blocked or shrunken, the behavior differs strongly depending on the dye, with the most favorable adsorption kinetics following the trend MB > MO > OG. Note that, for OG, only a very small proportion of the total dye present in the initial solution (C_0_) is adsorbed at low adsorption contact times. For sample A-H_3_PO_4_, which contains a high volume of mesopores of different sizes, the behavior is, however, very similar for the three adsorbates. With A-ZnCl_2_, A-ST, and A-KOH (Figure 7), the behaviors are intermediate, consistent with the porous texture of these adsorbents in the micropore region. In any case, the results suggest that not only ionic size, but also some other factor must play a very significant role in the adsorption kinetics of the dyes.

Since the equilibrium adsorption study of each dye showed that the best adsorption performance occurs with samples A-ST and A-KOH, Figures S6 and S7 (Supplementary Materials) also depict, for comparative purposes, the adsorption isotherms determined using these two adsorbents for the three dyes. As expected, adsorption of the dyes at equilibrium conditions is also more favorable for MB than for MO and OG.

Comparison of the kinetic and equilibrium adsorption results of MB using A-ST and A-KOH can be observed in Figures S8 and S9. From these figures, it can be concluded that A-KOH shows the best adsorption performance of the two, although the differences are not very significant in terms of adsorption kinetics. A direct quantitative comparison of dye adsorption capacities reported for activated carbons in the literature is inherently difficult, due to the wide variability in precursors, activation routes, textural properties, and experimental conditions (initial concentration range, adsorbent dose, solution chemistry, and data treatment). Nevertheless, a meaningful benchmark can be established by comparison with closely related studies carried out under comparable batch adsorption protocols. In recent works by our research group [82,83], steam-activated carbons prepared from polymeric waste streams exhibited equilibrium uptake capacities of the same order of magnitude for MB, MO, and OG, while chemically activated carbons showed Langmuir monolayer capacities for MB in the 0.7–0.9 × 10^−3^ mol g^−1^ range. In this context, the adsorption capacities obtained in the present study for coconut-shell-derived activated carbons are fully consistent with those reported for other high-performance carbonaceous adsorbents, confirming that appropriate activation strategies enable lignocellulosic wastes to yield competitive materials for dye removal from water.

#### 2.6.2. Adsorption Mechanism of the Dyes

The adsorption behavior of the three dyes on carbonized products and activated carbons derived from CS can be rationalized by jointly considering textural properties, surface chemistry, molecular size, polarity, and the information provided by kinetic and equilibrium modeling. In general, adsorption performance improves markedly for carbons prepared by physical activation with CO_2_ or steam and by chemical activation with KOH or H_3_PO_4_, while methylene blue (MB) is consistently adsorbed more efficiently than methyl orange (MO) and orange G (OG). At first glance, this trend may appear counterintuitive, since adsorption involves successive steps of diffusion in solution, intraparticle diffusion within pores, and surface attachment, and the latter two stages are expected to favor smaller adsorbates. The present results, therefore, highlight the complex interplay between molecular properties and adsorbent structure in dye adsorption systems.

Equilibrium modeling provides key mechanistic insight. For carbonized samples, the poor fit of the Langmuir model and the prevalence of Freundlich-type behavior indicate heterogeneous, low-energy surfaces where adsorption proceeds without well-defined monolayer saturation. This is consistent with their limited porosity and restricted accessibility of adsorption sites. In contrast, activated carbons—particularly A-CO_2_, A-ST and A-KOH—show excellent agreement with the Langmuir model for most dyes, indicating that adsorption is dominated by monolayer formation on a finite number of accessible sites. The simultaneous relevance of the Freundlich model, especially for A-H_3_PO_4_, reflects surface heterogeneity and the contribution of pores of different sizes, allowing coexistence of multiple adsorption environments.

Electrostatic interactions further modulate adsorption. MB carries a positive charge, whereas MO and OG are anionic, with OG containing two sulfonate groups. These charges influence both the orientation of the dye molecules and their affinity toward carbon surfaces of different acid–base character. However, electrostatics alone cannot explain the superior adsorption of MB, particularly on basic carbons, pointing to the dominant role of non-electrostatic interactions.

A more comprehensive descriptor of adsorbate behavior is the topological polar surface area (TPSA) [84,85], which increases in the order MB (43.9 Å^2^) < MO (93.5 Å^2^) < OG (176 Å^2^) [86]. High TPSA values promote strong hydration in aqueous solution, effectively increasing the apparent size of the adsorbate and hindering diffusion into narrow pores. Hydrated dye ions likely retain part of their solvation shell upon adsorption, limiting close contact with the surface and favoring adsorption through the non-polar aromatic moieties. Consequently, adsorption is mainly driven by π–π interactions between the aromatic rings of the dyes and the graphene-like domains of activated carbons.

The Langmuir-dominated behavior observed for the best-performing carbons supports a mechanism initially involving monolayer adsorption through these π–π interactions. Because MB occupies a smaller surface area per molecule than MO and OG, higher surface coverage is attainable, explaining its superior adsorption capacity. At higher equilibrium concentrations, the sharp increase in adsorption observed experimentally can be attributed to cooperative adsorbate–adsorbate interactions, leading to multilayer formation, as reflected by Freundlich behavior in specific systems.

To sum up, the combined kinetic and equilibrium analyses indicate that dye adsorption is governed not solely by ionic size, but by a balance between hydration effects, surface polarity, aromatic interactions, and pore accessibility. In this context, TPSA provides a more appropriate framework than bare ionic dimensions to interpret the adsorption trends observed for MB, MO, and OG.

## 3. Materials and Methods

### 3.1. Materials and Reagents

Coconut shell (CS) was used as a raw precursor. Fresh coconuts were purchased from local supermarkets in Badajoz (Extremadura, Spain). The shells were manually separated, washed, air-dried, crushed in a mechanical–manual mill, and sieved. The fraction with particle size greater than 1 mm was selected for carbon preparation. Elemental analysis (C, H, N, S) and ash content of the raw precursor were determined as described in Section 3.3.

Nitrogen (N_2_, N–48 quality), synthetic air (Alphagaz, Madrid, Spain), and CO_2_ were supplied by Air Liquide (Mérida, Spain). The activating agents used for chemical activation were H_3_PO_4_ (85%, Panreac, Barcelona, Spain), ZnCl_2_ (85%, Panreac), and KOH (85%, Panreac). KBr (Merck, Madrid, Spain, spectroscopy grade) was used for FTIR pellet preparation. The dyes used as model adsorbates—Methylene Blue (MB, C_16_H_18_ClN_3_S), Methyl Orange (MO, C_14_H_14_N_3_NaO_3_S), and Orange G (OG, C_16_H_10_N_2_Na_2_O_7_S_2_)—were purchased from Sigma Aldrich (Madrid, Spain). Deionized water was used in all experiments.

### 3.2. Preparation of Activated Carbons

#### 3.2.1. Carbonization

CS was carbonized in a Carbolite Gero horizontal tubular furnace HC (Neurtek S.L., Eibar, Spain) under N_2_ flow. Approximately 20 g of CS were placed in a ceramic boat, purged with N_2_ at 100 mL min^−1^, and heated from room temperature to either 600 or 900 °C at a rate of 10 °C min^−1^. The final temperature was maintained for 2 h, after which the samples were allowed to cool to room temperature under N_2_. The obtained were labeled C-600 and C-900. Several batches were carbonized to obtain sufficient material for the subsequent activation steps.

#### 3.2.2. Physical Activation

Physical activation was performed on C-600 and C-900 using air, CO_2_, or steam as activating agents. All activations were conducted in horizontal tubular furnaces under controlled gas flow following the same general protocol as in carbonization. Air activation was conducted at 400 °C for 1.3 h using C-600 as a starting material. CO_2_ activation was performed at 900 °C for 2.0 h using C-900. Steam activation was carried out with C-900 at 800 °C for 1.3 h in an Iberlabo two-furnace system (Iberlabo S.L., Madrid, Spain), in which water was vaporized at 250 °C and the resulting steam passed over the char placed in the activation furnace operating between 750 and 900 °C. Before the steam introduction, the system was purged with N_2_ for approximately 10 min. The physically activated carbons were coded A-O_2_, A-CO_2_, and A-ST.

#### 3.2.3. Chemical Activation

Chemical activation was carried out using H_3_PO_4_, ZnCl_2_, and KOH as activating agents (AA). CS was impregnated by wet mixing with aqueous activating solutions. In all cases, an activating agent–to–precursor mass ratio of 6:1 was used. For H_3_PO_4_ activation, approximately 20 g of CS were mixed with concentrated H_3_PO_4_ solution under magnetic stirring at 85 °C for 2 h in a covered beaker to minimize evaporation. For ZnCl_2_ and KOH activation, impregnations were carried out in a three-necked flask equipped with a reflux condenser and mechanical stirring, maintaining the slurry at 85 °C for 7 h in the case of ZnCl_2_ and for 2 h in the case of KOH. After impregnation, the solids were recovered by vacuum filtration and dried at 120 °C.

Carbonization/activation of the impregnated samples (~10 g per batch) was conducted in the Carbolite furnace under a N_2_ flow of 80 mL min^−1^. For H_3_PO_4_- and ZnCl_2_-impregnated samples, the temperature was increased to 500 °C at a heating rate of 10 °C min^−1^ and held for 2 h. For KOH-impregnated samples, the final temperature was 750 °C, and the residence time was also 2 h.

After carbonization/activation, the samples were washed to remove residual activating agents. ZnCl_2_-activated materials were washed sequentially with 5 M, 3 M, and 1 M HCl solutions, followed by 1 h treatment with 1 M HCl under stirring and subsequent rinsing with distilled water until neutral pH. KOH-activated materials were contacted once with 0.5 M HCl for 30 min and then repeatedly rinsed with distilled water until neutral pH. H_3_PO_4_-activated materials were washed repeatedly with distilled water to neutral pH. All washed samples were dried at 120 °C for 12 h, cooled in a desiccator over CaCl_2,_ and weighed to determine yield. The chemically activated carbons were coded A-H_3_PO_4_, A-ZnCl_2_, and A-KOH. All samples were stored in hermetically sealed containers until characterization and adsorption experiments.

### 3.3. Characterization

#### 3.3.1. Elemental Analysis and Ash Content

The elemental compositions (C, H, N, S) of raw CS and all carbon materials were determined using a LECO CHNS-932 microanalyzer by the Research Support Service of the University of Extremadura (SAIUEx, Badajoz, Spain). Oxygen content was calculated by difference. Ash content of raw CS was determined by heating approximately 1 g of sample in a muffle furnace at 650 °C for 12 h in air. Analyses were performed in triplicate.

#### 3.3.2. X-Ray Diffraction and X-Ray Fluorescence

Powder X-ray diffraction (XRD) patterns were recorded using a D8 Advance Bruker diffractometer (Bruker AXS, Karlsruhe, Germany). Elemental composition by wavelength-dispersive X-ray fluorescence (WDXRF) was determined using an S8 Tiger spectrometer (Bruker AXS, Karlsruhe, Germany) (Rh tube, up to 4 kW) with SPECTRAplus V3 software (SAIUEx).

#### 3.3.3. Scanning Electron Microscopy (SEM)

Surface morphology of the carbons was examined using a Quanta 3D FEG (FEI Company, Hillsboro, OR, USA) scanning electron microscope (SAIUEx), and representative micrographs were recorded from different surface regions of each sample.

#### 3.3.4. N_2_ Adsorption–Desorption at −196 °C

Textural properties were determined from N_2_ adsorption–desorption isotherms at −196 °C using an Autosorb-1 (Quantachrome, Boynton Beach, FL, USA) analyzer (SAIUEx). Approximately 0.15 g of sample was dried at 120 °C for 12 h, cooled in a CaCl_2_ desiccator, and degassed under vacuum (<10^−3^ Torr) at 120 °C for 12 h before analysis. The BET specific surface area was calculated from the isotherms using standard models.

#### 3.3.5. Mercury Porosimetry and Density

Macro- and mesoporosity were evaluated by mercury intrusion porosimetry using an Autoscan-60 (Quantachrome) porosimeter (SAIUEx) in the pressure range 0.10–414 MPa (pore diameters approximately 730,000 to 18 Å). Approximately 0.5 g of pre-dried sample was placed in a glass penetrometer, evacuated to 100 μm, and filled with mercury. The intruded volume was used to derive pore structure and density values.

#### 3.3.6. FTIR Spectroscopy

FTIR spectra of CS, chars, and activated carbons were recorded using a Perkin Elmer 1720 spectrophotometer (Waltham, MA, USA) in the range 400–4000 cm^−1^ with 2 cm^−1^ resolution and eight scans. KBr pellets were prepared by mixing the carbon sample with dry KBr in a mass ratio of 1:475 (total mass 238 mg) and pressing at 10 T cm^−2^ for 7–10 min. A KBr blank spectrum was used as background.

#### 3.3.7. Point of Zero Charge (pH_pzc_)

The pH_pzc_ was determined by the pH-drift method [87] using 0.01 M NaCl solutions initially adjusted to pH values between 2 and 10 with 0.1 M HCl or NaOH. For each measurement, approximately 0.10 g of carbon was placed in screw-cap tubes with 5.0 mL of solution and equilibrated in a thermostatic orbital shaker (25 °C, 50 rpm) for 48 h. After equilibration and filtration, the final pH was measured. The pH_pzc_ corresponded to the point where pH_final_ equaled pH_initial_.

#### 3.3.8. Yields of Chemical Activations

Given that the overall preparation of the activated carbon by the chemical activation method involves several steps in which mass gains or losses occur, the yield corresponding to each of these steps has been estimated separately, as well as the overall yield of the sample preparation process. These yields were calculated as follows:

- Yield of the impregnation treatment of CS:

(7) $$R_{I}=\frac{M_{IP}}{M_{CS}}\cdot 100$$

- Yield of the carbonization/activation process of the impregnated products:

(8) $$R_{A}=\frac{M_{AC}}{M_{IP}}\cdot 100$$

- Yield of the overall activated carbon preparation process:

(9) $$R_{P}=\frac{M_{AC}}{M_{CS}}\cdot 100$$

where M_CS_ is the mass of CS, M_IP_ the mass of the impregnated product (after impregnation and oven drying), and M_AC_ the mass of activated carbon (after carbonization of each impregnated product, washing with distilled water, and oven drying the resulting carbonized product at 120 °C).

### 3.4. Batch Adsorption Experiments

Batch adsorption studies were carried out using aqueous solutions of MB, MO, and OG. In the batch adsorption experiments, different experimental protocols were adopted for kinetic and equilibrium studies. For kinetic measurements, the same mass of adsorbent was used in all experiments to ensure direct comparability of adsorption rates and kinetic parameters among the different samples. In contrast, for the equilibrium isotherm experiments, the amount of adsorbent was varied in a controlled manner, which is a common and established practice in adsorption studies, in order to span a sufficiently wide range of equilibrium concentrations and to accurately determine isotherm parameters. This approach minimizes experimental bias and ensures reliable characterization of adsorption equilibria.

#### 3.4.1. Kinetic Studies

A known mass of adsorbent (~0.10 g for all materials except 0.015 g for A-KOH samples) was added to 25 mL screw-cap tubes containing 20 mL of dye solution of known initial concentration (1.41 × 10^−3^ mol L^−1^ for MB, 1.37 × 10^−3^ mol L^−1^ for MO, and 1 × 10^−3^ mol L^−1^ for OG). The tubes were placed in a thermostated shaker bath at 25 °C with 50 oscillations min^−1^. Contact time varied from 5 min to 528 h. At each predetermined time, a tube was removed from the bath, the suspension was filtered, and the residual dye concentration was determined as described in Section 3.5. The equilibrium time was obtained from the evolution profiles of dye concentration as a function of time.

#### 3.4.2. Equilibrium Isotherms

Equilibrium isotherms were obtained at 25 °C by varying the mass of adsorbent between 0.002 and 0.30 g (0.008–0.015 g for A-KOH samples) in 20 mL dye solution at the same initial concentrations used for kinetic experiments. The contact time was set to exceed the equilibrium time. After equilibration and filtration, the equilibrium concentration (C_e_) was determined, and the adsorbed amount (q_e_, mg g^−1^) was calculated by mass balance.

### 3.5. Analytical Determination of Dyes

The concentrations of MB, MO, and OG were measured by UV–Vis spectrophotometry using a Shimadzu UV-3101PC instrument (Shimadzu Corporation, Kyoto, Japan) and 1 cm path-length glass cuvettes. The maximum absorption wavelengths were 664 nm for MB, 464 nm for MO, and 477 nm for OG. Calibration curves were obtained from standard solutions, and the Lambert–Beer law was applied.

## 4. Conclusions

This study confirms that coconut shell (CS) is an excellent renewable precursor for activated carbon due to its high C and H contents and very low S and ash levels. Carbonization at 600 and 900 °C produced carbonaceous solids with increased C content but negligible porosity, demonstrating the need for activation to generate functional adsorbents.

Physical activation in air, CO_2_, and steam produced materials with low ash content and highly differentiated textural development. CO_2_ and steam yielded the most favorable textures (S_BET_ = 618 and 738 m^2^ g^−1^, respectively), characterized by broad microporosity and moderate mesoporosity, whereas air activation generated predominantly macroporosity. All physically activated carbons showed surface oxygen functionalities (O–H, C=O, C–O), with basic pH_pzc_ values (>8) for A-CO_2_ and A-ST and moderately acidic behavior for A-O_2_.

Chemical activation using ZnCl_2_, H_3_PO_4_, and KOH produced carbons with very high porosity development, especially A-H_3_PO_4_ (S_BET_ = 1798 m^2^ g^−1^, V_mi_ = 0.60 cm^3^ g^−1^) and A-KOH (S_BET_ = 1600 m^2^ g^−1^, V_mi_ = 0.70 cm^3^ g^−1^). Surface functionality profiles were similar across chemically activated samples, although their pH_pzc_ values were lower (<6), indicating independent control of porosity and surface chemistry.

Adsorption experiments with methylene blue (MB), methyl orange (MO), and orange G (OG) demonstrated that the carbonized samples exhibit slow kinetics and low adsorption capacities, as a direct consequence of their poorly developed porosity. In contrast, the activated carbons showed markedly enhanced kinetic and equilibrium performance, with the best results obtained for samples A-ST, A-H_3_PO_4_, and A-KOH. For MB adsorption, retention efficiencies higher than 90% were achieved with A-ST (93.9%), A-H_3_PO_4_ (98.1%), and A-KOH (90.7%). In the case of methyl orange, all activated carbons A-ST, A-H_3_PO_4_, A-ZnCl_2_, and A-KOH exhibited removal efficiencies exceeding 80%. For orange G, adsorption efficiencies above 85% were obtained with A-H_3_PO_4_ (88.0%) and A-ST (85.1%). Across all systems, MB was preferentially adsorbed (MB > MO > OG), a trend that correlates more strongly with the dyes’ topological polar surface area (TPSA)—43.9 Å^2^ (MB), 93.5 Å^2^ (MO), and 176 Å^2^ (OG)—than with ionic size. The lower polarity of MB leads to weaker hydration, facilitating diffusion into micropores and allowing a smaller adsorption footprint. The characteristic shape of the adsorption isotherms indicates an initial monolayer formation followed by multilayer adsorption, promoted by π–π interactions between the aromatic structures of the dyes and the graphene-like domains of the activated carbons.

Overall, the results demonstrate that the activation route dictates both the textural and surface chemistry properties of CS-derived activated carbons and, consequently, their adsorption performance. Steam-activated (A-ST) and KOH-activated (A-KOH) samples constitute the most promising materials, combining extensive microporosity, moderate mesoporosity, and favorable surface chemistry. These findings highlight the potential of coconut shell valorization to produce high-performance activated carbons for sustainable removal of organic dyes from wastewater.

## Figures and Tables

**Table 2:** Elemental and proximate analysis (weight %). Samples: CS, Carbonized Samples, Physically Activated Carbons, and Chemically Activated Carbons.

Sample	C	H	N	O	S	Ash
CS	46.80	6.52	0.06	46.59	0.03	0.45
C-600	86.2	2.83	0.42	0.55		1.28
C-900	86.7	1.52	0.56	11.22		1.03
A-O_2_	70.3	2.32	0.36	27.29		0.80
A-CO_2_	88.7	1.03	0.54	9.73		0.79
A-ST	87.9	1.29	0.44	10.37		1.46
A-ZnCl_2_	82.4	2.50	0.30	14.80		0.94
A-H_3_PO_4_	79.5	2.23	0.39	17.88		3.34
A-KOH	77.1	1.68	1.68	19.54		2.32

**Table 3:** FT-IR spectrum. Band assignments for sample CS.

Wavenumber/cm^−1^	Vibration Mode *	Functional Group/Assignment
3429	ν(O–H)	–OH (hydroxyl groups)
2924, 2873	ν(C–H)	–CH_3_, –CH_2_–
1729	ν(C=O)	Ketone, aldehyde, acid, ester
1633	ν(C=C)	Aliphatic/aromatic ring
1499, 1447	ν(C=C)	Aromatic
1447, 1381	δ(C–H)	–CH_3_, –CH_2_–
	δ(O–H), ν(C=O)	Carbonyl
1247	ν(=C–O–C)	Ether
1062	ν(C–O)	Ester, ether, alcohol
900–700	γ(C–H)	Aromatic
700–400	ν(C–C)	

* ν = stretching vibration; δ = in-plane deformation; γ = out-of-plane deformation.

**Table 4:** Textural parameters and pH_pzc_ values. Samples: Carbonized Samples, Physically Activated Carbons, and Chemically Activated Carbons.

Sample	Method/Overall Yield (%) *	S_BET_ /m^2^ g^−1^	W_0_ /cm^3^ g^−1^	V_mi_ /cm^3^ g^−1^	V_me_ /cm^3^ g^−1^	V_me-p_ /cm^3^ g^−1^	V_ma-p_ /cm^3^ g^−1^	ρ_Hg_ /cm^3^ g^−1^	V_T_ /cm^3^ g^−1^	pH_pzc_
C-600	Carbonized 33.0	7	0.01	0.00	0.00	0.05	0.06	1.31	0.12	—
C-900	Carbonized 23.2	129	0.07	0.06	0.04	0.00	0.00	1.07	0.07	—
A-O_2_	Physical 40.2	270	0.12	0.13	0.06	0.00	0.11	1.37	0.25	5.25
A-CO_2_	Physical 63.2	618	0.33	0.32	0.08	0.06	0.08	1.11	0.47	8.25
A-ST	Physical 52.2	738	0.39	0.38	0.07	0.01	0.01	1.11	0.41	9.25
A-ZnCl_2_	Chemical 28.3	992	0.53	0.50	0.07	0.08	0.07	1.01	0.68	4.44
A-H_3_PO_4_	Chemical 22.3	1798	0.60	0.64	0.61	0.44	0.16	0.64	1.20	2.49
A-KOH	Chemical 3.0	1600	0.70	0.74	0.22	0.00	0.85	0.33	1.55	5.80

* For chemically activated samples, the overall yield of the activated carbon preparation process; for physically activated samples, the yield corresponds to the activation stage; for carbonized products, the yield corresponds to carbonization.

**Table 5:** FT-IR spectra. Band assignments. Samples: C-600 and C-900.

Wavenumber/cm^−1^	Vibration Mode *	Functional Group/Assignment
3500	ν(O–H)	–OH (hydroxyl groups)
2932, 2847	ν(C–H)	–CH_3_, –CH_2_–
1729	ν(C=O)	Carboxylic acid
1607, 1486	ν(C=C)	Aliphatic/aromatic ring
1499, 1447	ν(C=C)	Aromatic
1447, 1389	δ(C–H)	–CH_3_, –CH_2_–
	δ(O–H), ν(C=O)	Carbonyl
1280	ν(=C–O)	Phenolic –OH
1123, 1068	ν(C–O)	Ether-type structures
900–700	γ(C–H)	Aromatic
700–400	ν(C–C)	

* ν = stretching vibration; δ = in-plane deformation; γ = out-of-plane deformation.

**Table 6:** Yields (weight %). Samples: A-ZnCl_2_, A-H_3_PO_4_, and A-KOH.

Sample	R_I_	R_A_	R_P_
A-ZnCl_2_	116	24.4	28.3
A-H_3_PO_4_	155	14.4	22.3
A-KOH	161	1.08	3.04

## Data Availability

The original contributions presented in this study are included in the article/Supplementary Material. Further inquiries can be directed to the corresponding authors.

## Supplementary Materials

The following supporting information can be downloaded at: https://www.mdpi.com/article/10.3390/molecules31020263/s1. Figure S1: N_2_ adsorption isotherms at −196 °C. Samples: A-O_2_, A-CO_2_, and A-ST; Figure S2: Mercury intrusion curves. Samples: A-O_2_, A-CO_2_ and A-ST; Figure S3: N_2_ adsorption isotherms at −196 °C. Samples: A-ZnCl_2_, A-H_3_PO_4_, and A-KOH; Figure S4: Mercury intrusion curves. Samples: A-ZnCl_2_, A-H_3_PO_4_ and A-KOH; Figure S5. Structures of MB, MO, and OG in their ionic forms; Figure S6: Adsorption isotherms of MB, MO, and OG. Sample: A-ST; Figure S7: Adsorption isotherms of MB, MO, and OG. Sample: A-KOH; Figure S8: MB adsorption. Kinetics. Samples: A-ST and A-KOH; Figure S9: MB adsorption. Isotherms. Samples: A-ST and A-KOH; Table S1: WDXRF analysis. Sample: CS ashes; Table S2: MB adsorption. Kinetic and equilibrium data; Table S3: MB adsorption. Parameters of the Langmuir and Freundlich equations; Table S4: MO adsorption. Kinetic and equilibrium data; Table S5: MO adsorption. Parameters of the Langmuir and Freundlich equations; Table S6: OG adsorption. Kinetic and equilibrium data; Table S7: OG adsorption. Parameters of the Langmuir and the Freundlich equations.

## Abbreviations

The following abbreviations are used in this manuscript:

A-CO_2_	Physically activated carbon with CO_2_
A-ST	Physically activated carbon with steam
A-H_3_PO_4_	Chemically activated carbon with H_3_PO_4_
A-KOH	Chemically activated carbon with KOH
A-O_2_	Physically activated carbon with O_2_
A-ZnCl_2_	Chemically activated carbon with ZnCl_2_
C-600	Char heated to 600 °C
C-900	Char heated to 900 °C
CS	Coconut shell
MB	Methylene Blue
MO	Methyl Orange
OG	Orange G
pH_pzc_	Point of zero charge
R_A_	Yield of the carbonization/activation process of the impregnated products
R_I_	Yield of the impregnation treatment of CS
R_P_	Yield of the overall activated carbon preparation process
SAIUEx	Research Support Service of the University of Extremadura, Spain
S_BET_	Specific surface area, calculated by the Brunauer–Emmett–Teller (BET) adsorption model
SEM	Scanning electron microscopy
TPSA	Topological polar surface area
V_ma-p_	Macropore volume (mercury intrusion)
V_me_	Mesopore volume (N_2_ adsorption isotherm)
V_me-p_	Mesopore volume (mercury intrusion)
V_mi_	Micropore volume (N_2_ adsorption isotherm)
V_T_	Total pore volume
W_0_	Micropore volume (N_2_ adsorption isotherm, Dubinin)
WDXRF	Wavelength-dispersive X-ray fluorescence
XRD	Powder X-ray diffraction
ρ_Hg_	Density of mercury
