# Supplementary material for: Coconut Shell-Derived Activated Carbons: Preparation, Physicochemical Properties, and Dye Removal from Water
Source: Molecules. 2026 Jan 12;31(2):263. doi: 10.3390/molecules31020263 (PMC12844380; doi:10.3390/molecules31020263)
Supplement: Supplementary file 1 [file molecules-31-00263-s001.zip › molecules-4044718-supplementary.pdf]

Supplementary Materials

for

Coconut Shell–Derived Activated Carbons: Preparation, Physicochemical Properties, and Dye Removal from Water

Vanda María Cachola Maldito Lowden, María Francisca Alexandre-Franco *, Juan Manuel Garrido-Zoido *, Eduardo Manuel Cuerda-Correa and Vicente Gómez-Serrano

Departamento de Química Orgánica e Inorgánica, Facultad de Ciencias, Universidad de Extremadura, Avenida de Elvas s/n, 06006 Badajoz, Spain; vanda_cachola@hotmail.com (V.M.C.M.L.); emcc@unex.es (E.M.C.-C.); vgomez@unex.es (V.G.-S.)
* Correspondence: malexandre@unex.es (M.F.A.-F.); jmgarridoz@unex.es (J.M.G.-Z.)

Figures: 9

Tables: 7

Table S1. WDXRF analysis. Sample: CS ashes.

Formula	Z	Concentration
K_2O	19	41.36 %
Na_2O	11	14.00 %
SiO_2	14	6.18 %
P_2O_5	15	4.67 %
SO_3	16	3.58 %
CaO	20	3.13 %
MgO	12	2.92 %
Cl	17	1.38 %
Al_2O_3	13	0.60 %
ZnO	30	0.48 %
Fe_2O_3	26	0.45 %
CuO	29	0.29 %
Rb_2O	37	0.10 %
MnO	25	527 ppm
SrO	38	503 ppm
NiO	28	140 ppm
Br	35	122 ppm

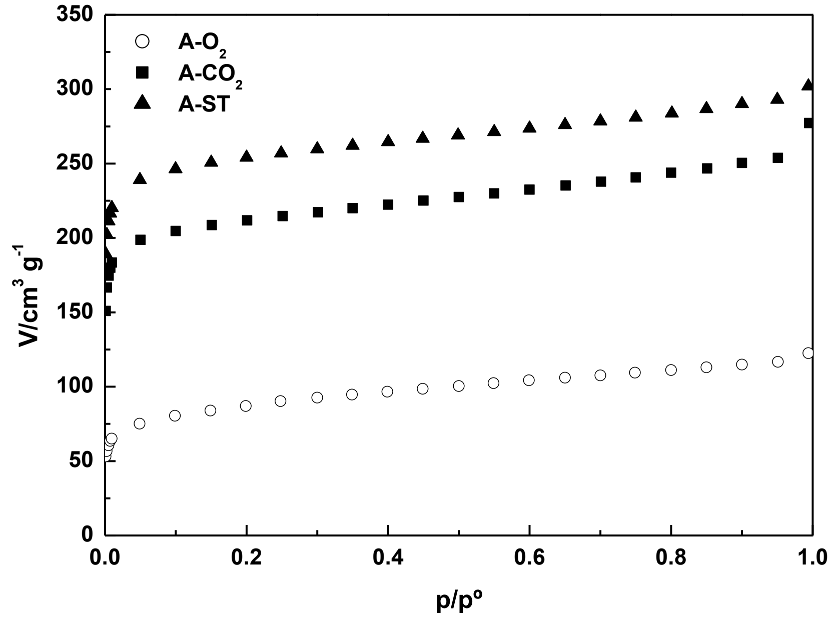

Figure S1. N_2 adsorption isotherms at −196 °C. Samples: A-O_2, A-CO_2 and A-ST.

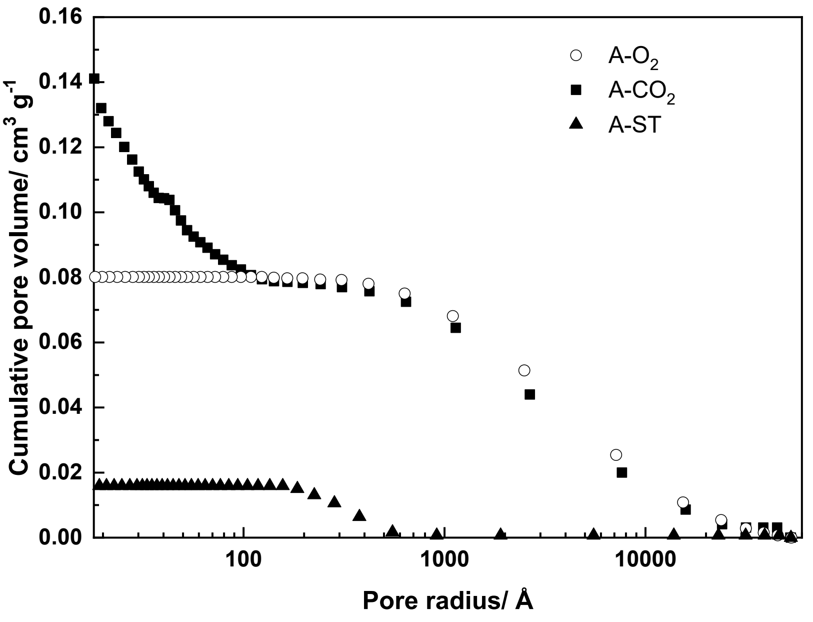

Figure S2. Mercury intrusion curves. Samples: A-O_2, A-CO_2 and A-ST.

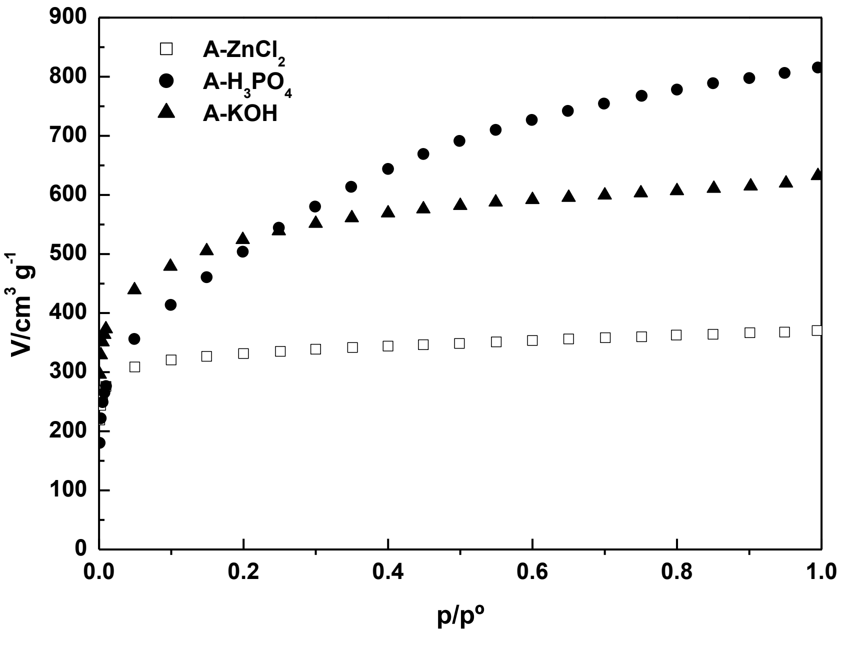

Figure S3. N_2 adsorption isotherms at −196 °C. Samples: A-$ZnCl_2$, A-H_3PO_4 and A-KOH.

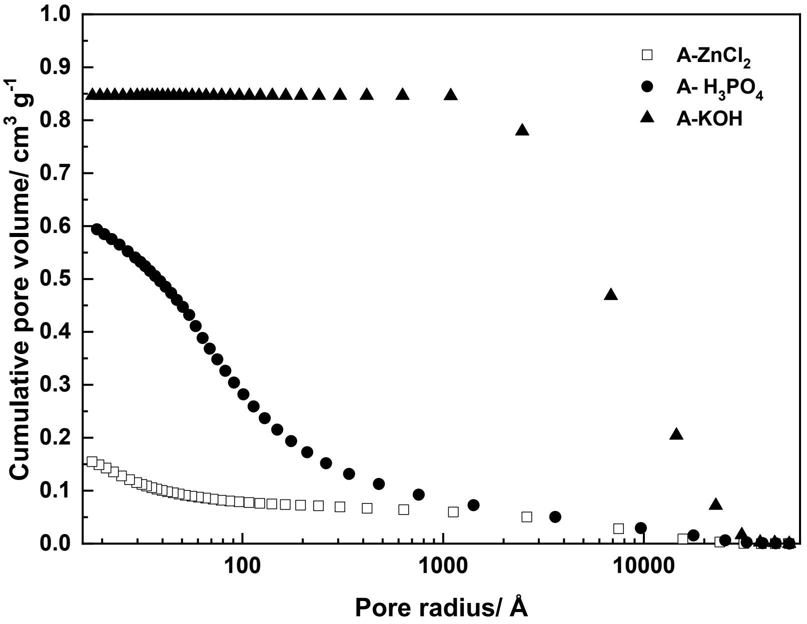

Figure S4. Mercury intrusion curves. Samples: A-$ZnCl_2$, A-H_3PO_4 and A-KOH.

Table S2. MB adsorption. Kinetic and equilibrium data.

			Pseudo-first order kinetic model			Pseudo-second order kinetic model		
**Sample**	**t_e (h)**	**$q_e×10^4$ (experimental)**	**$q_e×10^4$ (mol·g^{-1})**	**$k_1×10^2$ (h^{-1})**	**R^2**	**$q_e×10^4$ (mol·g^{-1})**	**$k_2×10^{-3}$ (g·mol^{-1}·h^{-1})**	**R^2**
C-600	50	1.67	0.80	0.10	0.902	1.69	0.06	0.999
C-900	150	2.50	1.66	4.58	0.961	2.57	1.02	0.993
A-O_2	50	1.84	0.47	0.11	0.986	1.82	0.40	0.999
A-CO_2	200	2.47	0.85	3.13	0.972	2.49	2.27	0.999
A-ST	200	2.65	1.44	3.16	0.984	2.67	1.05	0.999
A-$ZnCl_2$	400	2.58	2.15	0.90	0.994	2.71	0.15	0.985
A-H_3PO_4	75	2.54	2.23	11.2	0.956	2.78	0.69	0.999
A-KOH	75	17.1	6.49	4.51	0.945	17.2	0.47	0.999

Table S3. MB adsorption. Parameters of the Langmuir and Freundlich equations.

	Langmuir				Freundlich		
**Sample**	**$Q_0×10^3$ (mol·g^{-1})**	**b×10^{-3} (L·mol^{-1})**	**$R_L×10^4$**	**R^2**	**1/n**	**$K_F×10^2$ (mol·g^{-1}/(mol·L^{-1})$^{1/n}$)**	**R^2**
C-600	0.26	1.60	4.40	0.760	0.80	5.00	0.902
C-900	0.26	4.33	1.60	0.947	0.85	15.00	0.703
A-O_2	0.58	1.28	5.50	0.893	0.70	5.00	0.989
A-CO_2	0.52	30.84	2.00	0.987	0.24	0.30	0.984
A-ST	0.48	54.86	1.00	0.999	0.20	0.20	0.980
A-$ZnCl_2$	0.43	12.92	0.60	0.963	0.20	0.15	0.898
A-H_3PO_4	0.77	5.44	7.70	0.677	0.61	6.72	0.907
A-KOH	2.14	7.39	0.10	0.986	0.34	2.29	0.887

Table S4. MO adsorption. Kinetic and equilibrium data.

			Pseudo-first order kinetic model			Pseudo-second order kinetic model		
**Sample**	**t_e (h)**	**q_e×10^4 (experimental)**	**q_e×10^4 (mol·g^{-1})**	**k_1×10^2 (h^{-1})**	**R^2**	**q_e×10^4 (mol·g^{-1})**	**k_2×10^{-2} (g·mol^{-1}·h^{-1})**	**R^2**
C-600	25	0.21	0.05	1.47	0.118	0.24	84.2	0.985
C-900	150	1.73	1.34	2.63	0.844	2.21	5.29	0.992
A-O_2	50	0.35	0.20	5.53	0.986	0.40	162	0.999
A-CO_2	350	2.62	1.78	1.01	0.961	2.67	2.61	0.992
A-ST	350	2.26	2.12	1.22	0.985	2.46	1.73	0.980
A-$ZnCl_2$	250	2.29	1.39	1.52	0.913	2.33	6.38	0.998
A-H_3PO_4	75	2.37	1.69	5.46	0.979	2.45	7.45	0.999
A-KOH	75	15.6	7.37	8.54	0.956	15.1	5.58	0.999

Table S5. MO adsorption. Parameters of the Langmuir and Freundlich equations.

	Langmuir				Freundlich		
**Sample**	**Q_0×10^3 (mol·g^{-1})**	**b×10^{-3} (L·mol^{-1})**	**R_L×10^4**	**R^2**	**1/n**	**K_F×10^3 (mol·g^{-1}/(mol·L^{-1})$^{1/n}$)**	**R^2**
C-600	—	—	—	0.907	59.71	∞	0.885
C-900	0.37	18.98	—	0.946	0.22	1.74	0.975
A-O_2	—	—	—	0.876	4.57	∞	0.870
A-CO_2	0.29	20.88	0.40	0.994	0.30	2.75	0.932
A-ST	0.29	24.72	0.30	0.991	0.24	1.70	0.965
A-$ZnCl_2$	0.24	7.33	1.00	0.998	0.36	2.67	0.985
A-H_3PO_4	699.15	0.11	4114.00	0.998	0.29	1126.94	0.873
A-KOH	1.27	118.95	0.10	0.900	0.26	8.30	0.929

Table S6. OG adsorption. Kinetic and equilibrium data.

			Pseudo-first order kinetic model			Pseudo-second order kinetic model		
**Sample**	**t_e (h)**	**q_e×10^5 (experimental)**	**q_e×10^5 (mol·g^{-1})**	**k_1×10^2 (h^{-1})**	**R^2**	**q_e×10^5 (mol·g^{-1})**	**k_2×10^{-4} (g·mol^{-1}·h^{-1})**	**R^2**
C-600	50	1.0	1.19	0.15	0.756	1.17	2.63	0.999
C-900	50	1.96	1.09	50.3	0.983	1.91	0.3	0.999
A-O_2	50	1.22	0.74	8.13	0.895	1.35	2.47	0.999
A-CO_2	350	13.7	12.5	2.95	0.992	13.8	73.8	0.996
A-ST	350	13.1	12.4	82.9	0.985	19.6	328	0.990
A-$ZnCl_2$	200	10.0	10.0	2.35	0.978	11.8	170	0.993
A-H_3PO_4	150	17.5	17.2	3.57	0.917	17.9	604	0.993
A-KOH	150	72.8	35.0	4.63	0.867	73.6	750	0.998

Table S7. OG adsorption. Parameters of the Langmuir and Freundlich equations.

Sample	Langmuir				Freundlich		
	$Q_0 \times 10^3$ (mol·g^{-1})	$b \times 10^{-3}$ (L·mol^{-1})	$R_L \times 10^4$	R^2	1/n	$K_F \times 10^3$ (mol·g^{-1}/(mol·L^{-1})$^{1/n}$)	R^2
C-600	—	—	—	0.542	4.07	∞	0.700
C-900	0.57	0.30	34.00	0.693	0.92	78.0	0.972
A-O_2	0.03	3.82	3.00	0.576	28.82	∞	0.870
A-CO_2	0.13	113.98	0.01	0.990	0.16	0.50	0.887
A-ST	0.14	160.83	1.00	0.986	0.15	0.40	0.956
A-$ZnCl_2$	0.22	12.52	1.00	0.928	0.26	1.40	0.900
A-H_3PO_4	0.23	54.25	0.20	0.979	0.18	0.90	0.980
A-KOH	0.91	65.28	0.20	0.821	0.95	778.0	0.853

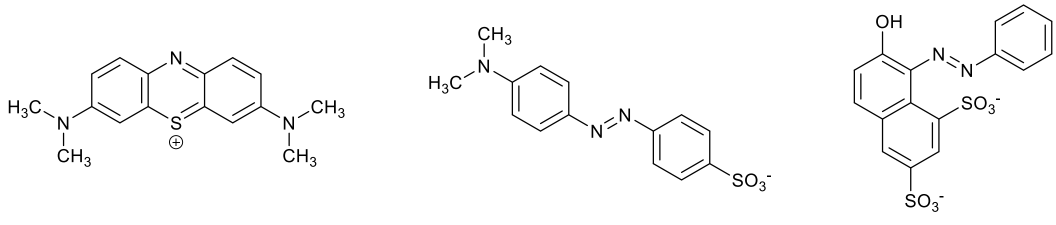

MB **MO** **OG**

Figure S5. Structures of MB, MO, and OG in their ionic forms.

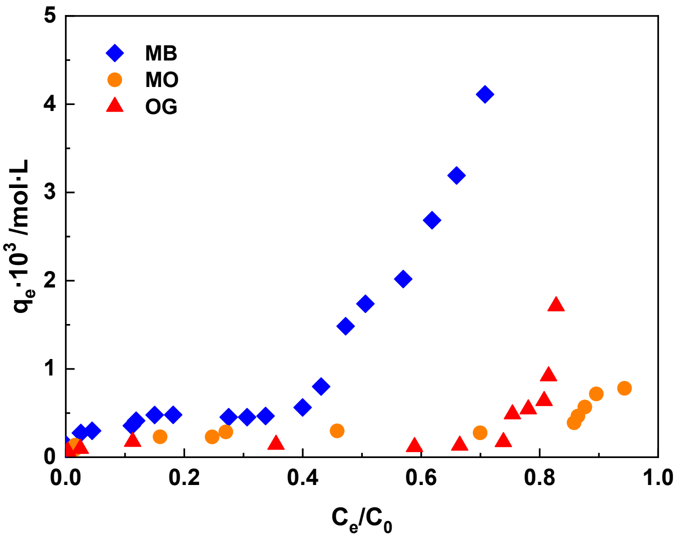

Figure S6. Adsorption isotherms of MB, MO, and OG. Sample: A-ST.

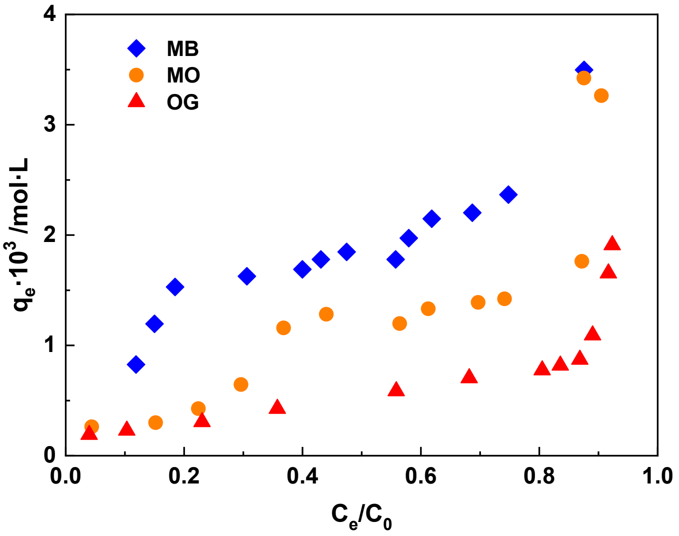

Figure S7. Adsorption isotherms of MB, MO, and OG. Sample: A-KOH.

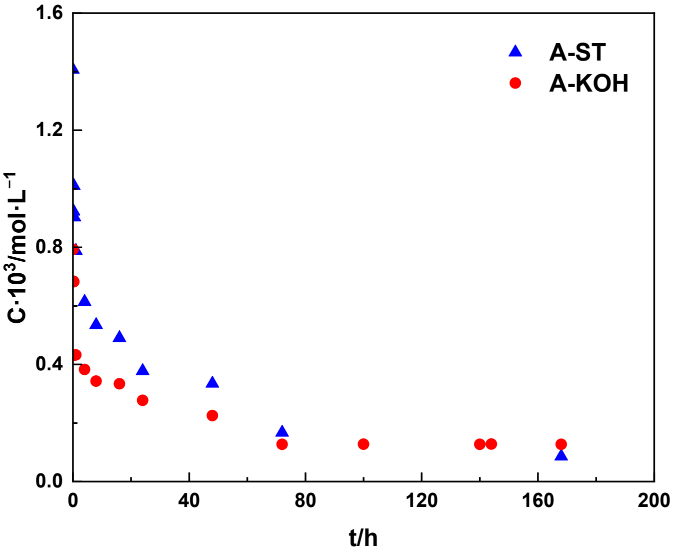

Figure S8. MB adsorption. Kinetics. Samples: A-ST and A-KOH.

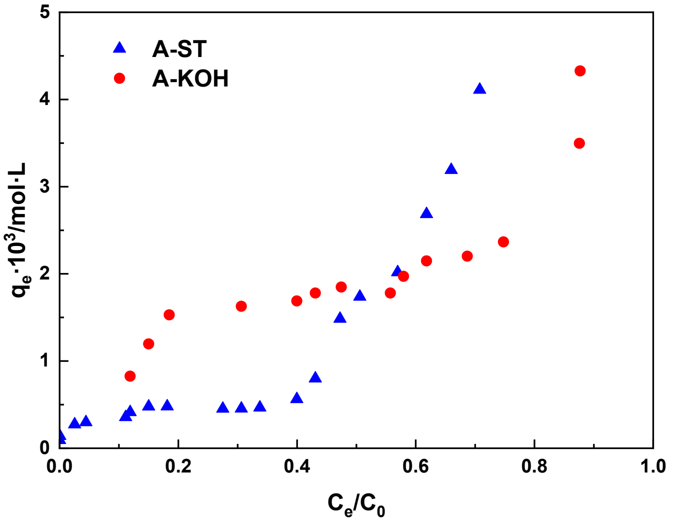

Figure S9. MB adsorption. Isotherms. Samples: A-ST and A-KOH.
